# Identification and functional analysis of three new anthocyanin R2R3‐MYB genes in *Petunia*

**DOI:** 10.1002/pld3.114

**Published:** 2019-01-21

**Authors:** Hechen Zhang, Ronald Koes, Hongquan Shang, Zhenzhu Fu, Limin Wang, Xiaoyu Dong, Jing Zhang, Valentina Passeri, Yanbang Li, Hui Jiang, Jie Gao, Yanmin Li, Huijuan Wang, Francesca M. Quattrocchio

**Affiliations:** ^1^ Horticulture Research Institute Henan Academy of Agricultural Sciences Zhengzhou China; ^2^ Department of Plant Development and (Epi) Genetics Swammerdam Institute for Life Sciences University of Amsterdam Amsterdam The Netherlands; ^3^ Department of Plant Science School of Agriculture and Biology Shanghai Jiao Tong University Shanghai China

**Keywords:** anthocyanin synthesis, evolution, *Petunia*, R2R3‐MYBs, transcription factor

## Abstract

We identified three novel members of the R2R3‐MYB clade of anthocyanin regulators in the genome of the purple flowering *Petunia inflata S6* wild accession, and we called them *ANTHOCYANIN SYNTHESIS REGULATOR* (*ASR*). Two of these genes, *ASR1* and *ASR2,* are inactivated by two different single base mutations in their coding sequence. All three of these genes are absent in the white flowering species *P. axillaris N* and *P. parodii*, in the red flowering *P. exserta*, and in several *Petunia hybrida* lines, including R27 and W115. *P. violacea* and other *P*. *hybrida* lines (M1, V30, and W59) instead harbor functional copies of the *ASR* genes. Comparative, functional and phylogenic analysis of anthocyanin R2R3‐MYB genes strongly suggest that the *ASR* genes cluster is a duplication of the genomic fragment containing the other three R2R3‐MYB genes with roles in pigmentation that were previously defined, the *ANTHOCYANIN4‐DEEP PURPLE*‐*PURPLE HAZE* (*AN4*‐*DPL*‐*PHZ*) cluster. An investigation of the genomic fragments containing anthocyanin MYBs in different *Petuni*a accessions reveals that massive rearrangements have taken place, resulting in large differences in the regions surrounding these genes, even in closely related species. Yeast two‐hybrid assays showed that the ASR proteins can participate in the WMBW (WRKY, MYB, B‐HLH, and WDR) anthocyanin regulatory complex by interacting with the transcription factors AN1 and AN11. All three ASRs can induce anthocyanin synthesis when ectopically expressed in *P. hybrida* lines, but ASR1 appeared to be the most effective. The expression patterns of *ASR1* and *ASR2* cover several different petunia tissues with higher expression at early stages of bud development. In contrast, *ASR3* is only weakly expressed in the stigma, ovary, and anther filaments. The characterization of these novel *ASR* MYB genes completes the picture of the MYB members of the petunia anthocyanin regulatory MBW complex and suggests possible mechanisms of the diversification of pigmentation patterns during plant evolution.

## 1 INTRODUCTION

Pigmentation is a suitable model to study how new patterns are generated during the evolution of species. The study of regulatory genes involved in the formation of spots on *Drosophila* wings revealed how mutations contributed to the diversification of sexual preference and thus to genetic separation and the appearance of new species (Gompel, Prud'homme, Wittkopp, Kassner, & Carroll, 2005). The diversification of the coat color in mammals has been shown to be related to fitness in different environments (Hoekstra, 2006), and the understanding of the mechanism behind such variation is a key to the unraveling of adaptation mechanisms. In plants, the formation of different pigmentation patterns is often related to reproduction since many species display color to attract animals for the dispersal of pollen and seeds (Galliot, Stuurman, & Kuhlemeier, 2006) but also contribute to the adaptation to different growth conditions (Albert et al., 2014; Anderson, Willis, & Mitchell‐Olds, 2011; Steyn, Wand, Holcroft, & Jacobs, 2002). The most widely diffused plant pigments are anthocyanins. Their biosynthesis is one of the best‐studied metabolic pathways, making it very attractive to use these pigments as a model to understand how patterns are generated during the evolution of a species.

Anthocyanins are flavonoid pigments providing blue/violet pigmentation to foliage, fruit, and flowers and they fulfill a variety of physiological functions (Tanaka, Sasaki, & Ohmiya, 2008; Winkel‐Shirley, 2001). The synthesis of anthocyanins is regulated by a network of transcription factors determining tissue specificity and response to the stimuli of the pigment accumulation. In all species examined to date, these transcription factors are R2R3‐MYB, bHLH, and WD40 proteins forming an MBW protein complex, which activates the promoters of the anthocyanins synthesis structural genes (Spelt, Quattrocchio, Mol, & Koes, 2002; Koes, Verweij, & Quattrocchio, 2005; Ramsay & Glover, 2005; Gonzalez, Zhao, Leavitt, & Lloyd, 2008; ;Albert et al., 2014). The complex is boosted by the participation of a WRKY transcription factor, which also confers specificity for other sets of target genes involved in, for example, vacuolar hyperacidification (Verweij et al., 2016). The WDR (WD40) regulators are highly conserved, even among animals and plants (de Vetten, Quattrocchio, Mol, & Koes, 1997). To date, a single gene in all species, *Transparent Testa Glabra1* (*TTG1*) in Arabidopsis, *PAC* in maize and *AN11* in petunia, is known to encode the WD40 member of the MBW complex (Carey, Strahle, Selinger, & Chandler, 2004; de Vetten et al., 1997; Walker et al., 1999).

The bHLH anthocyanin regulators can instead be grouped into at least two phylogenetic clades, and most species have members belonging to each clade. One group includes maize B, Lc, and R (Purugganan & Wessler, 1996), petunia JAF13 (Quattrocchio, Wing, van der Woude, Mol, & Koes, 1998), and Arabidopsis GL3 and EGL3 (Bernhardt et al., 2003). The Arabidopsis TT8 protein groups in a distinct clade (Consonni, Geuna, Gavazzi, & Tonelli, 1993; Hernandez, Feller, Morohashi, Frame, & Grotewold, 2007) together with the petunia AN1 (Spelt, Quattrocchio, Mol, & Koes, 2000) and the maize IN factor (Burr et al., 1996). The WRKY factor is encoded by a single gene in all studied species (Amato et al., 2017; Johnson, Kolevski, & Smyth, 2002; Verweij et al., 2016). All components of the WMBW complex are essential to efficiently activate anthocyanin synthesis, as shown by the loss (or reduction) of pigmentation in mutants for each of these factors.

R2R3‐MYB components of the WMBW complex determine the set of target genes that the complex will activate. The gene families are classified into several subgroups with different functions in plant‐specific processes, such as development, signal transduction, resistance to pathogens, and metabolism (including anthocyanin synthesis) (Dubos et al., 2010). The members of the group represented by the petunia AN2 is characterized by the R2R3‐MYB Sub‐Group 6 (called SG6), sharing a short amino acid signature for anthocyanin regulating MYBs (Stracke, Werber, & Weisshaar, 2001; Zimmermann, Heim, Weisshaar, & Uhrig, 2004). SG6 MYBs are encoded in each species by a small family of genes with different expression patterns, contributing to the color of different plant parts (Albert et al., 2011; Gonzalez et al., 2008; Quattrocchio et al., 1998; Schwinn et al., 2006). The spotted pattern of the petals of *Lilium* hybrids has been shown to be associated with *LhMYB6* or *LhMYB12* (Yamagishi, Shimoyamada, Nakatsuka, & Masuda, 2010), while in *Clarkia*, the expression domain of an SG6 MYB, *CgMYB1*, determines the position of a spot in the flower (Martins, Berg, Blinka, Rausher, & Baum, 2013; Martins, Jiang, & Rausher, 2017). These examples show that duplication and diversification of R2R3‐MYB genes controlling anthocyanin synthesis result in different expression patterns of these regulators, leading to considerable possibilities of variation in temporal and spatial anthocyanin accumulation in plants (Albert et al., 2011; Bombarely et al., 2016; Schwinn et al., 2006).

*Petunia hybrida* has a long history as a genetic model system (Vandenbussche, Chambrier, Rodrigues Bento, & Morel, 2016), particularly in the genetics of pigmentation. The MYB member of the WMBW complex regulating anthocyanin accumulation in petunia was thought to be one of four MYBs: *AN2*,* AN4*,* DEEP PURPLE* (*DPL*), and *PURPLE HAZE* (*PHZ*). All of these genes encode similar proteins but differ in expression patterns controlling pigmentation in distinct tissues or under different conditions. *AN2* is expressed in the petal limb and tube, whereas *AN4* is expressed in the anthers at early developmental stages (Supplemental note in Bombarely et al., 2016). *PHZ* is a light‐regulated MYB gene, whereas *DPL* regulates venation patterning in petunia flowers (Albert et al., 2011, 2014). These R2R3‐MYBs determine the timing, localization, and degree of anthocyanin accumulation.

In this study, we characterized three novel petunia R2R3‐MYB genes of the SG6 clade, *ASR1* to *ASR3*, and examined their roles in regulating anthocyanin accumulation and their possible involvement in creating pigmentation patterns in petunia. ASRs, similar to other SG6 MYB members, are also involved in the WMBW regulatory complex, where they interact with AN1 and AN11 and contribute to anthocyanin synthesis by upregulating anthocyanin structural genes.

## 2 MATERIALS AND METHODS

### 2.1 Plant materials and growing conditions

The petunia species *Petunia axillaris N S26*,* P. inflata S6*,* P. exserta S25*,* P. parodii S8*,* P. violacea S9*, and all of the *Petunia hybrida* lines used in this work (Supporting Information Table S1) come from the collection of the University of Amsterdam. Plants were grown at standard greenhouse conditions. As the M1 and R27 lines are not easily transformable, they were crossed to generate M1×R27/F1 progeny, which performed well in the production of stable transformants.

### 2.2 Identification and phylogenetic analysis of *ASR* genes in *Petunia*

The *ASR* genes were identified by screening the genome of *Petunia axillaris N* and *Petunia inflata S6* with known plant SG6 R2R3‐MYB sequences by BLAST search. The entire candidate sequences identified in this search were then used in a phylogenetic analysis, and *ASR1*,* ASR2,* and *ASR3* were selected because they clustered in the clade of other well characterized SG6 MYBs. Sequence alignments were generated via multiple sequence alignment using DNAMAN software, and phylogenetic trees were constructed on‐line (http://www.phylogeny.fr/index.cgi) based on multiple alignments of DNA or predicted amino acid sequences. The sequences of Arabidopsis genes were obtained from TAIR and those of other plants from other databases as referred to by the accession numbers. The accession numbers of all genes or proteins appearing in this paper are shown in Supporting Information Table S3.

### 2.3 Synteny analysis of genomic fragment containing MYBs cluster

All of the genomic data of *Solanaceae* and *Petunia* were mined from the Sol Genomics Network (https://solgenomics.net/). We manually annotated some of the genes.

### 2.4 Generation of constructs

All constructs in this study were made using the Gateway cloning system. The cDNA or gDNA of *ASR* genes and other genes were amplified by PCR and the fragments were inserted into entry vectors (pDONR207, pDONR221) by BP reaction. The entry vectors were then used in recombination reactions with the destination vectors pK2GW2.0/rfa (OE), pKGWFS7.0/rfa (Promoter:GUS), pGBKT7/GW (Y2H), and pGADT7/GW (Y2H) by LR reaction. All constructs are described in Supporting Information Table S4.

### 2.5 Petunia transformation

Constructs were transformed into *Agrobacterium tumefaciens* strain *Agl1*. Stable transformants of *Petunia hybrida* M1×R27 or W115 were generated by *Agrobacterium*‐mediated leaf‐disc transformation. The regenerated plants were checked by PCR for the presence of the construct and by RT‐PCR for its expression.

### 2.6 pH measurement

pH measurements were performed as described previously (Verweij et al., 2008).

### 2.7 Determination of anthocyanin content by HPLC

HPLC analysis was carried out by Scistd Testing Co., LTD. (Qingdao, China). Anthocyanins were extracted using 50 mg of powdered petal (flower development stage 2–3) suspended in 5 ml of 0.5% (v/v) HCl‐methanol and incubated for 2 hr at 4°C in the dark. The extracts were then centrifuged for 15 min at 14,000 rpm, and the supernatants were collected and stored at −20°C. The supernatant was diluted with methanol to 5 ml and filtered through a cellulose acetate membrane (Sartorius, Göttingen, Germany). HPLC was performed with Agilent 1260 Infinity LC (Agilent Technologies, USA). Anthocyanidin profile analysis was quantified at the 530 nm wavelength by using a calibration curve from commercial standards of anthocyanidins (pelargonidin, cyanidin, delphinidin, petunidin, peonidin, and malvidin, Sigma, USA).

### 2.8 RT‐PCR and qRT‐PCR analysis

To determine the expression of the genes of interest, we analyzed their expression profiles by RT‐PCR or qRT‐PCR (Real‐time RT‐PCR) methods. Total RNA was extracted via the TRIzol method, and its quantity and quality was measured by Nanodrop (Thermo Fisher Scientific, USA). cDNA synthesis was performed with PrimeScript™ II 1st Strand cDNA Synthesis Kit (Takara, Japan) and RT‐PCR analysis was carried out as previously described (Zhang, Yin, & Xia, 2008). qRT‐PCR was performed using the Bio‐Rad CFX Connect (Bio‐Rad, USA) with a SYBR‐Green PCR Master Mix Kit (SYBR^®^ *Premix Ex Taq*™, Japan). Transcript abundance was calculated relative to *actin* by using the 2^−ΔΔ*Ct*^ method. The primers used for these PCR analyses are listed in Supporting Information Table S5.

### 2.9 Isolation of *ASR* genes promoters and GUS reporter studies

The promoters of the *ASR1*,* ASR2,* and *ASR3* genes from *P. inflata* S6 were amplified from genomic DNA using primers 323 and 325 (pASR1), 404 and 321 (pASR2), and 416 and 317 (pASR3). The promoters of *ASR1* and *ASR2* were isolated from the petunia M1 line using a Genome walking kit (Takara biomed, Takara, Japan) with gene specific primers and the universal primer AP4. The promoters were amplified and cloned in front of the *GUS* reporter gene in the pKGWFS7.0/rfa vector using the Gateway system. M1×R27F1 hybrid petunia plants were used as hosts for the generation of stable transformants. Histochemical GUS staining was performed as described previously (Albert et al., 2014). Petunia tissues were pretreated with diethyl ether for 30 s, immersed for 1 min in 90% ice cold acetone and then rinsed twice in 50 mM phosphate buffer (pH 7). The samples were then incubated in X‐gluc staining solution (with or without 1 mM K_3_Fe(CN)_6_/K_4_Fe(CN)_6_) overnight at 37°C. Seedlings were then decolored by treatment with 70% ethanol.

### 2.10 Yeast two‐hybrid assay

The BD or AD constructs for the expression of the fusion proteins in yeast were first transformed into the yeast strain AH109 and the yeast transformants were then screened by growth on SC‐Leu (for AD) or SC‐Trp (for BD) plates (2–3 days at 30°C). The transformant colonies that grew under these conditions were then used for cotransformation with the second construct for the interaction assay and plated on SC‐Leu/Trp media plates. Yeast double transformants were diluted in ddH_2_O, plated again on selective SC‐Leu/Trp/Ade/His medium at different dilutions (10×, 500×, and 5,000×), and grown for 2–4 days. All transformants containing BD‐ASR constructs displayed self‐activation (detectable by growth on selective medium also in the presence of an empty AD‐ vector). Therefore, only AD‐ASR constructs were used to test interactions with other proteins expressed as BD‐fusions.

## 3 RESULTS

### 3.1 Three novel members of the anthocyanin R2R3‐MYB family in *Petunia*

**Figure 1:**
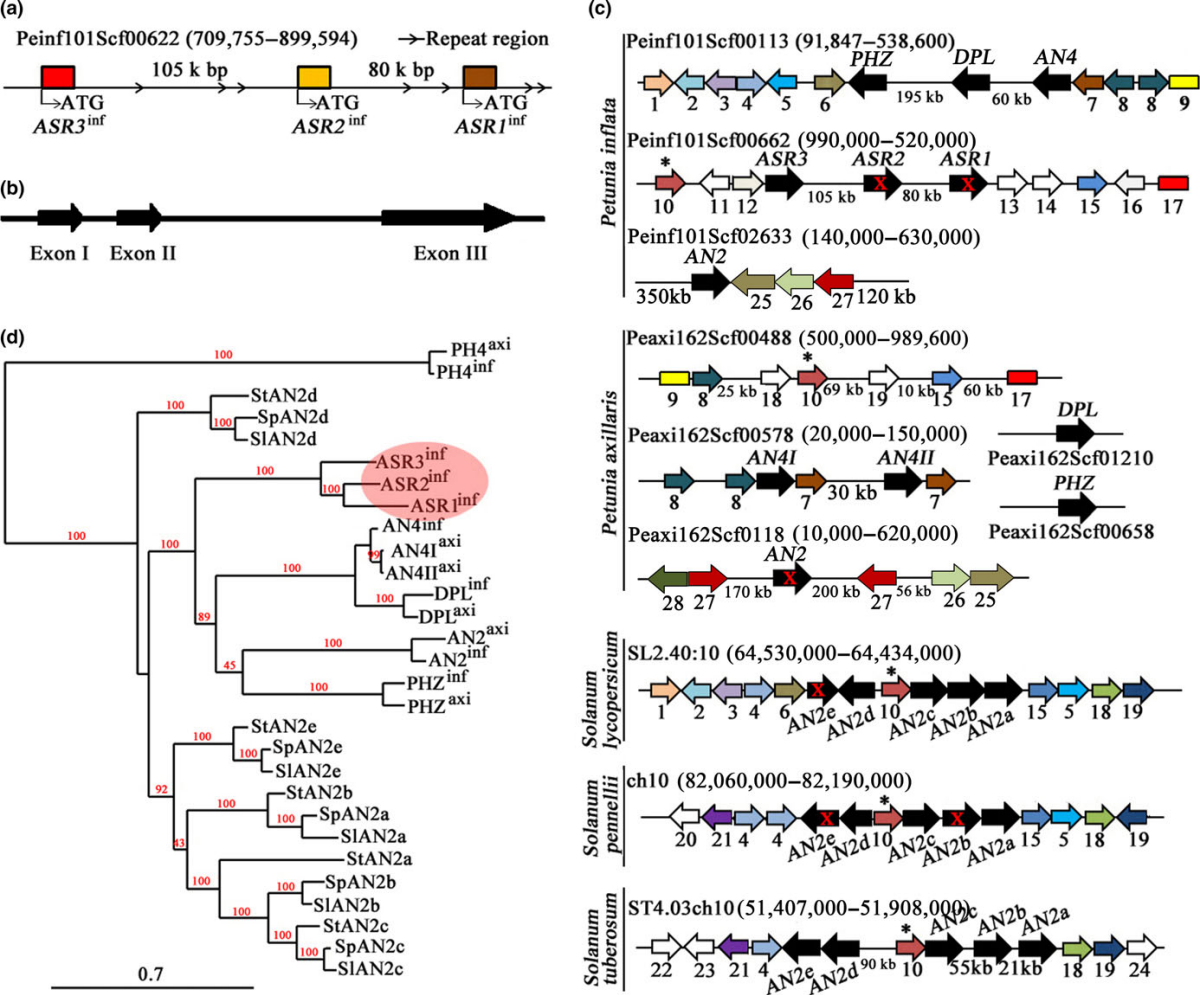
*ASR* genes cluster in *Petunia*. (a) The genomic fragment containing the *ASR* cluster in *P. inflata*. (b) Architecture of *ASR* genes in *P. inflata*. Exons are shown as arrow boxes. (c) Synteny analysis of the genomic regions containing SG6 MYB genes (black arrows) in *Petunia* and in three *Solanum* species. Arrows indicate genes and lines gene‐poor regions. Drawings are not to scale. Conserved genes are indicated by the same color. Red crosses indicate mutations in coding sequences that result in inactive proteins. The genes or sequences appearing here were summarized in Supporting Information Table S2. Among these genes, a much conserved gene (asterisk) for a heavy metal associated protein (10) is located in the genomic fragment in which SG6 genes are present in both *Petunia* and all *Solanum* species included in this analysis. (d) Phylogenetic analysis of the anthocyanin MYB genes in three *Solanum* and two *Petunia* species (genomic DNA sequences including introns). All of the *Solanum* genomic sequences were obtained from the SOL Genomic Network (https://solgenomics.net/). More information about the sequences used here is given in Supporting Information Table S3

R2R3‐MYB proteins, such as the petunia AN2, AN4, PHZ, and DPL (Albert et al., 2011; Quattrocchio et al., 1999), the Arabidopsis PAP1 and PAP2 (Maier et al., 2013; Teng, Keurentjes, Bentsink, Koornneef, & Smeekens, 2005), the grape VvMYBA1 and VvMYBA2 (Walker et al., 2007), and the apple MdMYB1 and 10 (Espley et al., 2007; Takos et al., 2006), have been demonstrated to play crucial roles in the regulation of anthocyanin synthesis. Searching the genomes of the petunia wild species *P. axillaris* and *P. inflata* for anthocyanin R2R3‐MYBs resulted in the identification of three new members of this clade in *P. inflata S6*, which are absent in *P. axillaris*. Because of their similarity to known R2R3‐MYB anthocyanin regulators, we named these three genes *ASR* (*ANTHOCYANIN SYNTHESIS REGULATOR*). In the genome of *P. inflata*, these genes are arranged in a cluster (present within a single scaffold) (Figure 1a). All three *ASR* genes have a structure identical to *AN2*,* AN4*,* DPL,* and *PHZ* containing two introns (Figure 1b). Comparison of the genomic regions containing these MYB clusters in tomato (*Solanum lycopersicum)*, wild tomato (*S. pennellii*), and potato (*S. tuberosum*), with *P. inflata* and *P. axillaris* scaffolds containing *AN2*,* AN4*‐*DPL*‐*PHZ,* and *ASR1*‐*ASR2*‐*ASR3* reveals some levels of shared synteny. The arrangement of the genes surrounding the MYB genes in all of these species supports their common origin suggested by the results of the phylogeny analysis and implicating a series of duplications that have generated the numerous members of this family in each *Solanacea* species. However, synteny analysis also suggests that many of these rearrangements took place in the short time separating these closely related species.

The two copies of *AN4* (and of some surrounding genes) in *P. axillaris* could be the reminiscence of the duplication that generated the *ASR1*,* ASR2*,* ASR3* cluster, which in this lineage was followed by further rearrangements leading to partial loss of the cluster. Even though both *P. axillaris* and *P. inflata* contain a copy of the *AN2* gene, these genes are contained in genomic fragments that were rearranged, resulting in a different distribution of surrounding genes in the two species. For example, on the right of *AN2*, the serine protease family protein (25), ARMADILLO/BETA‐CATENIN repeat family protein (26), and the serine/threonine protein kinase (27), are conserved, but their positions were rearranged. In addition, the *P. axillaris AN2* allele contains a mutation that destroys the coding sequence (Quattrocchio et al., 1999), whereas the *P. inflata* allele encodes a wild type protein (Figure 1c).

The phylogenetic relations among these R2R3‐MYBs in petunia and different species of the *Solanum* genus show that all anthocyanin MYB genes of petunia are originated by duplication events that happened in the *Petunia* genus itself (Figure 1d). This finding further supports the previously proposed scenario that describes the evolution of specific sets of anthocyanin R2R3‐MYBs by recent duplications occurred in each species independently, thus generating species specific sets of SG6 genes and, consequently, unique pigmentation patterns (Supplemental note in Bombarely et al., 2016).

### 3.2 *ASR1*‐*ASR2*‐*ASR3* cluster of anthocyanin regulators is different in distinct petunia accessions

**Figure 2:**
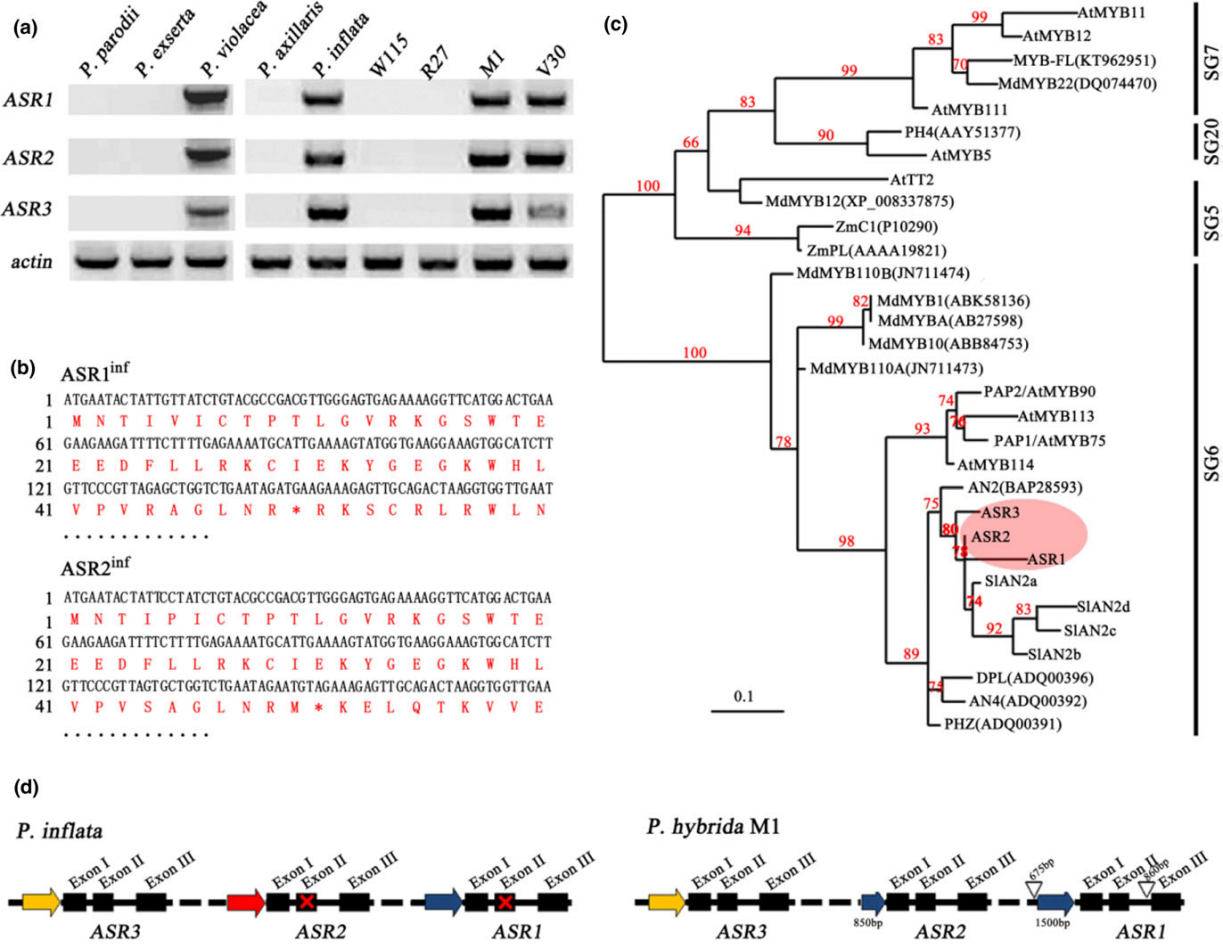
ASRs encode SG6 R2R3‐MYB proteins. (a) The *ASR* gene cluster is present in *P. inflata*,* P. violacea*, and in the *P. hybrida* lines M1 and V30 but is absent in *P. axallaris*,* P. parodii*,* P. exserta*, and in the *P. hybrida* lines W115, R27. A PCR method was used to amplify the specific ASR genes with their specific primers from DNA. (b) ASR1 and ASR2 encode truncated proteins in *P. inflata*. The mutations are indicated by an asterisk in the sequences. (c) Phylogenetic analysis of SG6 proteins in different species. The tree was constructed with maximum likelihood based on multiple alignments of the amino acid sequence of the R2R3‐motif only. (d) *ASR* genes from *P. inflata* and the *P. hybrida* line M1. Exons in *ASR* genes are indicated as solid boxes; similar promoter regions are shown as solid arrows of the same color, open triangles indicate insertions. Drawings are not to scale. Red crosses indicate mutation interrupting coding sequences

Analysis of different petunia wild species revealed that all three *ASR* genes are present in the *P. violacea* accession and all of the three (the complete cluster) are missing in *P. parodii* and *P. exserta*. Therefore, it is not surprising that some *Petunia hybrida* lines, generated by repeated crosses among different wild species, have the genomic fragments containing these three genes (M1, V30, W59) and others do not (W115 and R27) (Figure 2a, Supporting Information Table S1). In *P. inflata*, a one base mutation in *ASR1* ^inf^ at position 150 bp from the ATG and a one base insertion at position 148 bp in *ASR2* ^inf^ (Figure 2b), interrupt the reading frame in these two genes. However, these mutations are absent in the *ASR1* and *ASR2* alleles of the *P. hybrida* lines M1, V30, and W59, which encode full size proteins. This finding brings up the previously proposed possibility that other wild petunia species, in addition to *P. inflata* and *P. axillaris*, contributed to the genomes of the *P. hybrida* lines (Bombarely et al., 2016). We indeed found that the purple flowering wild accession *P. violacea* has two sets of *ASRs*: one copy of each gene is similar to the *P. inflata* allele, and the other is similar to that of the *P. hybrida* line M1. Because the *P. violacea* material that we used is highly inbred, it is improbable that the two sequences we obtained belong to different alleles of the same locus, and we therefore concluded that these genes are present in two copies. This finding suggests that a genome from the *P. violacea* lineage contributed to the crosses from which (some of) the *Petunia hybrida* lines were generated.

The phylogenic tree of the deduced amino acid sequences of ASRs and other R2R3‐MYBs in petunia further confirms that they encode SG6 MYBs and indicates that the different members of this group originate from duplications of a common ancestor and subsequent independent rearrangements in the different petunia species (Figure 2c). The comparison of the sequences of the *ASR1‐ASR2‐ASR3* cluster from *P. inflata* and the *P. hybrida* line M1 shows several rearrangements. As shown in Figure 2d, a comparison between the *ASR* genes in *P. inflata* and in the *P. hybrida* line M1 reveals a 675 bp insertion present in the *ASR1* ^M1^ promoter region, approximately 1,500 bp upstream of the start codon. The *ASR2* ^M1^ promoter contains instead an 850 bp fragment, similar to a fragment of the *ASR1* promoter. An 860 bp insertion is also found in the second intron of *ASR1* ^M1^.

### 3.3 ASR MYBs ectopic expression induces anthocyanin synthesis in different petunia organs

**Figure 3:**
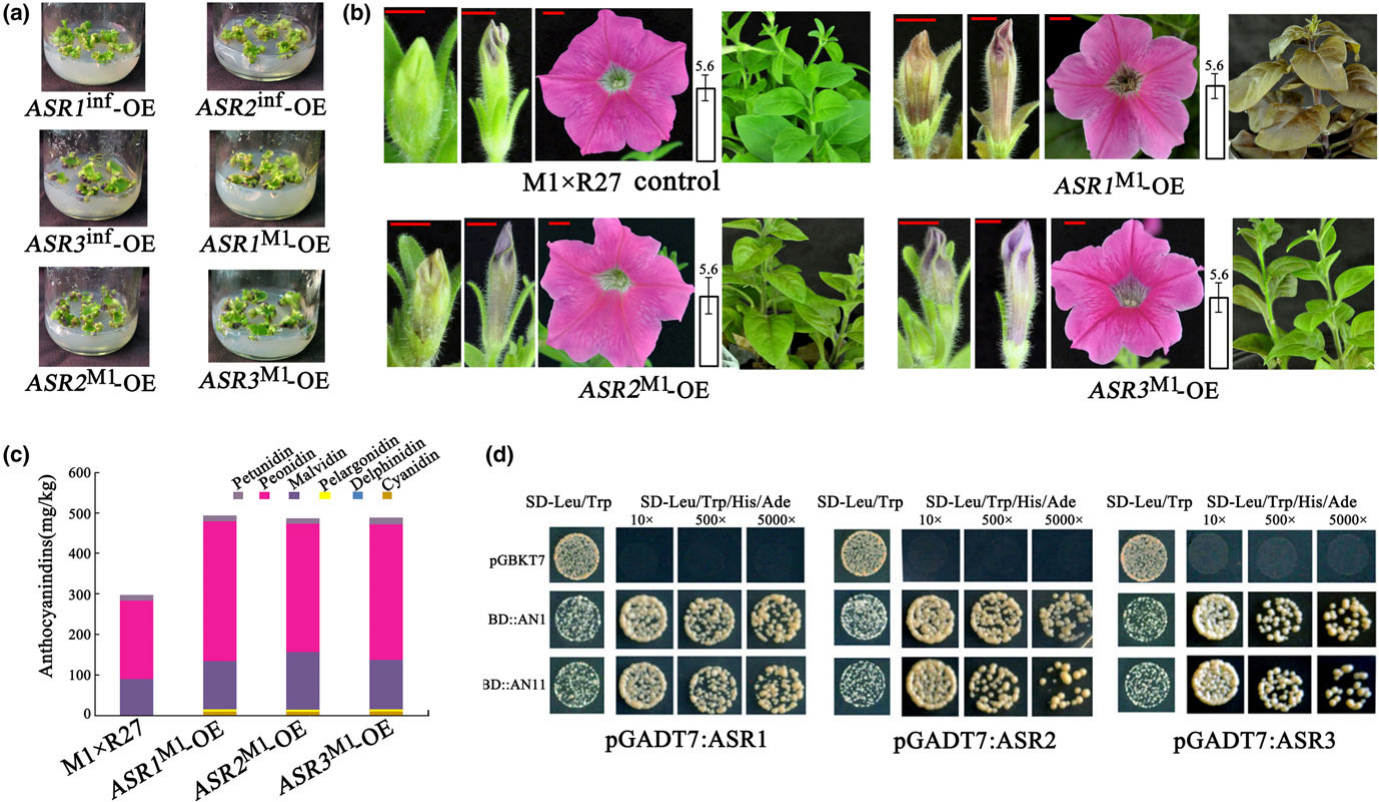
*ASR1* ^M1^, *ASR2* ^M1^, and *ASR3* ^*M1*^ all induce anthocyanin accumulation in different plant parts, and when ectopically expressed, are functionally similar. (a) Calli transformed with 35S:*ASR1* ^M1^ are strongly pigmented, whereas those transformed with 35S:*ASR1* ^inf^ (inactive allele) show no pigmentation. (b) Pigmentation induced by the expression of *ASRs* in seedlings of transgenic lines in the hybrid M1×R27 between two *Petunia hybrida* lines. Petal phenotype at development stage 2, 4, and 7. Red size bars equal 5 mm. On the right of each panel is the pH value of the crude petal extract at development stage 7 (after bud opening). (c) Anthocyanin accumulation in *ASRs* ^M1^‐OE petals at development stage 2–3, when no obvious color is yet visible in buds of untransformed controls. Anthocyanin levels are indicated as the mean of three biological replicates. Variation in biological replicates is less than 5%; therefore no error bar is shown here. (d) Interaction of ASRs^M1^ with AN1 and AN11 tested by a yeast two‐hybrid assay. The dilution series (10×, 500×, and 5,000×) were grown for 3 days before being photographed [Correction added on 31 January 2019, after first online publication: in the original version of Fig. 3a the same image was inadvertently used for ASR1^M1^‐OE and ASR2^M1^‐OE. This has now been amended.]

To test the role of *ASR* genes in anthocyanin synthesis, we generated *ASR1*,* 2,* and *3* overexpression (OE) lines containing constructs in which the coding sequences (from the *Petunia hybrida* line M1, *ASRs* ^M1^ or from *Petunia inflata*,* ASRs* ^inf^) are expressed from the *CaMV35S* promoter. As a host, we used the F1 hybrid between the two *P*. *hybrida* lines M1 and R27. Over 50 independent lines were generated for each construct. As shown in Figure 3a, even the calli of *ASR1* ^M1^‐OE, *ASR2* ^M1^‐OE, *ASR3* ^M1^‐OE, and *ASR3* ^inf^‐OE were pigmented, which was not observed for *ASR1* ^inf^‐OE and *ASR2* ^inf^‐OE as predicted because *ASR1* ^inf^ and *ASR2* ^inf^ encode truncated proteins. In the *ASRs* ^M1^‐OE lines, flower buds showed pigmentation in very early stages of floral development when buds of the untransformed control are completely acyanic. However, after the buds opened, pigmentation did not differ significantly from that of the untransformed control lines. *AN2* reaches high expression in petals shortly before bud opening (Quattrocchio et al., 1998), explaining why ectopic expression of the *ASR* genes induces early pigmentation of the young flower bud (when no *AN2* activity is present) but does not contribute to increasing it further in the open flowers. Although overexpression of each of the three *ASR* genes affected pigmentation, *ASR1* ^M1^ *‐*OE plants showed the most intense pigmentation, which included the stamens, stamen filaments, and gynoecium of the flower (Supporting Information Figure S2a). We could not measure obvious differences in the pH of the crude petal extract for flowers of any of the *ASR* ^M1^‐OE lines compared to untransformed controls, suggesting that ASRs do not regulate the structural genes involved in vacuolar hyperacidification (Faraco et al., 2014).

To further evaluate the effect of *ASR* expression on pigmentation, we measured the anthocyanidin content of flower petals at bud stage 2–3 in the overexpression lines. At this stage, no obvious coloration is detectable in buds of untransformed M1×R27 plants, while in transgenic plants, young buds are already colored. Petals of transgenic lines accumulate mainly peonidin and malvidin derivatives, similar to open buds of untransformed controls, which confer a magenta color to the tissue. *ASRs* ^M1^‐OE young petals have higher anthocyanidin content than petals of the same age in controls, as is already clear by visual investigation (Figure 3c). In conclusion, expression of *ASR* genes induces anthocyanin accumulation.

Several studies previously showed that R2R3‐MYBs of the SG6 clade interact with bHLH factors and, in this way, participate to form WMBW regulatory complexes. Alignment of the deduced protein sequences for ASR1, ASR2, and ASR3 (for the ASR1 and ASR2 alleles of *P. inflata*, we have manually corrected the coding sequence to restore the reading frame destroyed by the mutation) showed that all three of them, as well as AN2, AN4, DPL, and PHZ, contain the conserved [DL_x2_R_x3_L_x6_L_x3_] motif required for interactions with bHLH proteins (Supporting Information Figure S3) (Zimmermann et al., 2004). We used yeast two‐hybrid (Y2H) assays to examine the interaction of ASR1, ASR2, and ASR3 (from the *Petunia hybrida* line M1) with AN1 and AN11. This assay showed that the ASRs interact with both AN1 and AN11 (Figure 3d), implying that the ASR proteins can potentially participate in the WMBW complex that regulates anthocyanin biosynthesis.

### 3.4 ASR MYBs upregulate the expression of anthocyanin structural genes

**Figure 4:**
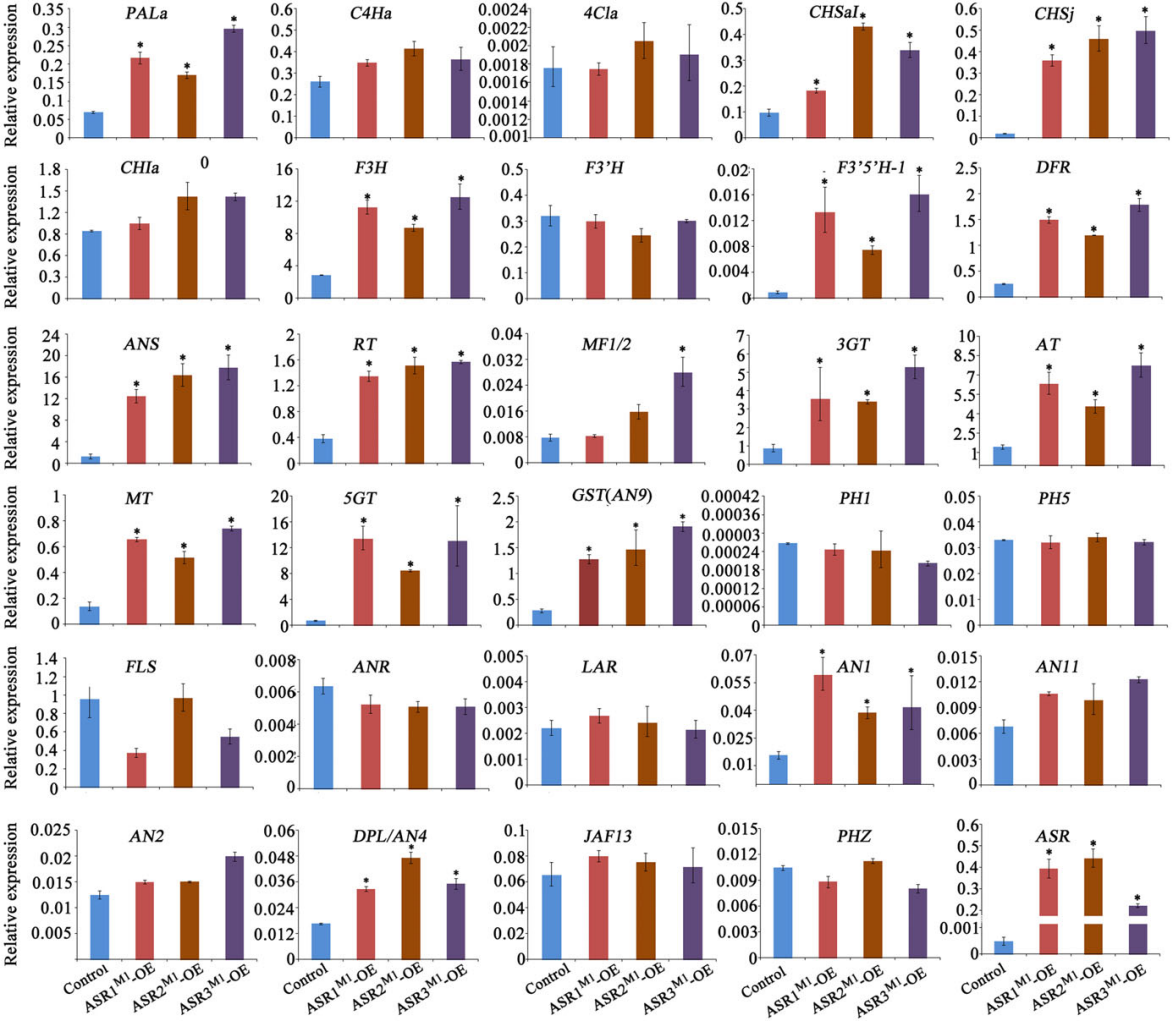
Expression of *ASRs* ^M1^ induced pigmentation genes. Real‐time RT‐PCR analysis of the expression of anthocyanin structural and regulatory genes as well as vacuolar hyperacidification genes in petals of buds at developmental stage 2–3 from the untransformed control (M1×R27), *ASR1* ^M1^‐OE,*ASR2* ^M1^‐OE, and *ASR3* ^M1^‐OE. Relative expression is indicated as the mean ± *SD* of three biological replicates. Statistical significance was determined by one‐way ANOVA and significant differences between means is indicated by an asterisk (*p*‐value < 0.05)

We compared the expression profiles of anthocyanin structural genes in young bud petals of untransformed M1×R27 hybrids and *ASRs* ^M1^‐OE lines by qRT‐PCR (Figure 4). Transcript levels of *PALa*,* CHSaI*,* CHSj*,* F3H*,* F3′5′H*,* DFR*,* ANS*,* RT*,* MT*,* 5GT*, and *AT* are increased in the overexpression lines for *ASRs* ^M1^ compared to untransformed controls. Among these genes, most of the so‐called Late Biosynthetic Genes (LBGs), operating in the pathway starting from the step controlled by DFR, are strongly activated by the ASR regulators. However, the expression of the EBGs (Early Biosynthetic Genes), *C4Ha*,* CHIa*,* 4CLa*, and *F3′H*, were not affected. The Methylation at Five (*MF*) gene (Provenzano et al., 2014) responds differently to the different *ASR* MYBs. The tannin‐related genes *ANR* and *LAR* were not affected by *ASRs* ^M1^ overexpression, indicating that these MYBs are specific for the anthocyanin pathway. Similarly, no expression of the flavonol synthase gene (*FLS)* was induced in *ASRs‐*OE lines, and no induction was observed for the expression of *PH1* and *PH5*, the two P‐ATPases required for vacuolar hyperacidification in the epidermis of petals (Faraco et al., 2014, 2017; Quattrocchio et al., 2006; Verweij et al., 2008), consistent with the pH values measured in petals.

**Figure 5:**
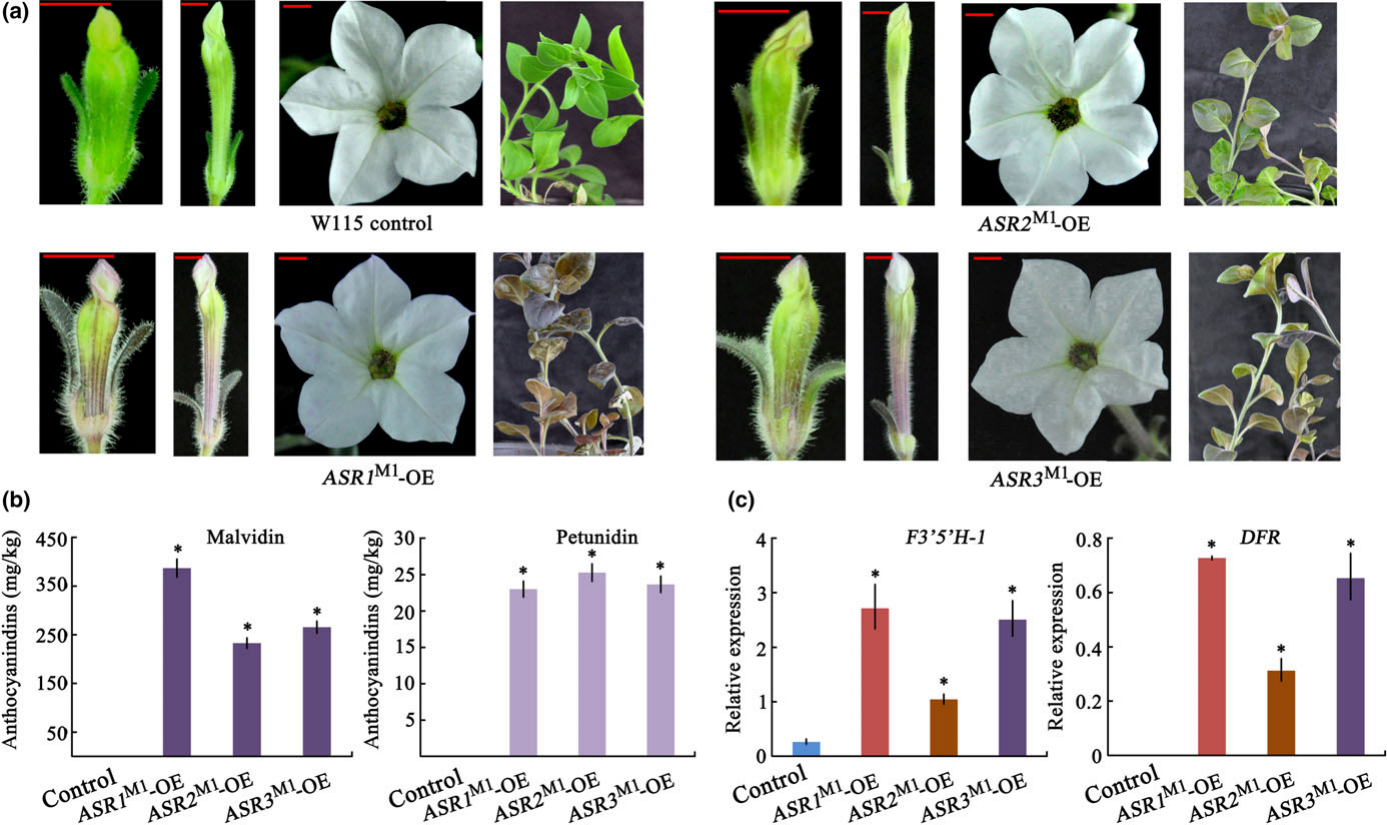
Overexpression of *ASRs* ^M1^ enhance anthocyanin levels in the *an2 an4* double mutant (*Petunia hybrida* line W115). (a) Flowers at development stages of 2, 4, and 7, and vegetative plant parts. Red size bars equal 5 mm. (b) Anthocyanin content in young leaves of untransformed control W115 and *ASRs* ^M1^‐OE plants indicated as the mean ± SD of three biological replicates. (c) Analysis of the expression of *F3′5′H* and *DFR* genes in young leaves of W115 untransformed control and *ASRs* ^M1^‐OE plant. Relative expression is indicated as the mean ± *SD* of three biological replicates. Statistical significance was determined by one‐way ANOVA and significant differences between means is indicated by an asterisk (*p*‐value < 0.05)

To test the effect of ASR MYBs in a background with reduced activity of other anthocyanin R2R3‐MYBs, we also overexpressed *ASR1* ^M1^, *ASR2* ^M1^, and *ASR3* ^M1^ in the W115 *Petunia hybrida* line. This mutant is a double *an2 an4* mutant that lacks the *ASR* cluster (similar to *P. axillaris*). Both petals and anthers of the flowers are acyanic in this line; therefore, the contribution of *ASR* genes to the pigmentation of flower organs can be more easily assessed in this genetic background. All lines ectopically expressing *ASRs* ^M1^ in W115 had dusky bronze vegetative organs; *ASR1* ^M1^‐OE plants were the most intensely pigmented and *ASR2* ^M1^‐OE plants the least (Figure 5a). We further observed that OE of *ASRs* ^M1^ increased pigmentation in young flower buds, especially at the early stages of floral development. Upon opening, however, all flowers were white, possibly because of the presence of the dominant allele of the *FADING* locus in W115 (Passeri, Koes, & Quattrocchio, 2016; de Vlaming & van Eekeres, 1982). The anthers of *ASR* ^M1^ *‐*OE plants were also (partially) pigmented, whereas the filaments and pistils did not differ in color from those of the untransformed controls. In addition, the seed pods in the early stages of development are pigmented in *ASRs* ^M1^ overexpression lines in this background, while in the M1×R27 background, this finding was not observed, probably due to the weak *hf1* allele (Supporting Information Figure S2). Analysis of anthocyanidin content in the young leaves of transgenic plants detected only highly substituted malvidin and petunidin derivatives, while no cyanidin‐related products were detected in these *ASR*‐OE plants, which was consistent with significant induction of expression of the *F3′5′H* gene (Figure 5b,c). We can conclude from this analysis that ASR1, ASR2, and ASR3 are indeed anthocyanin regulators sharing similar functions with other MYBs of the SG6 clade. However, although all of these genes are all effective in inducing anthocyanin accumulation, differences are detected in their effect on specific target genes.

### 3.5 *ASR* genes are driven by highly rearranged promoters that confer different expression patterns

**Figure 6:**
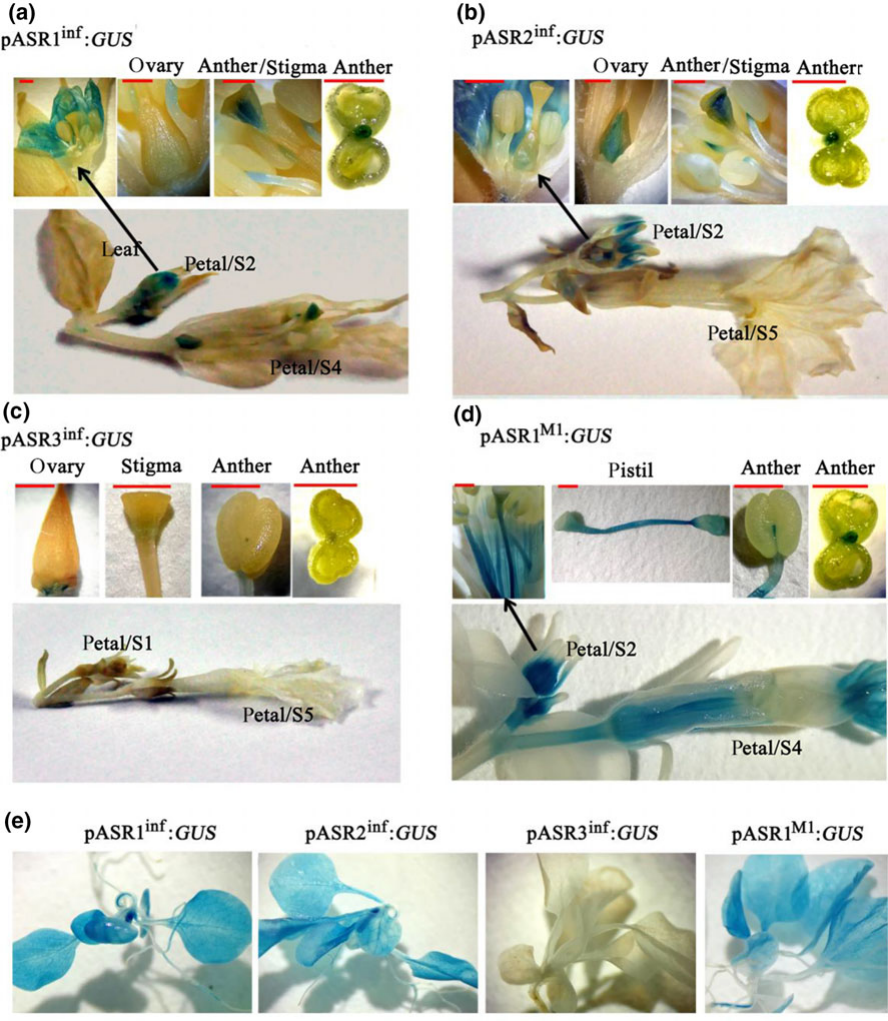
Promoter activity of *ASRs* in different petunia organs. Histochemical localization of GUS activity in flower organs from pASR1^inf^:GUS (a), pASR2^inf^:GUS (b), pASR3^inf^:GUS (c), and pASR1^M1^:GUS transgenic plants (d). Histochemical localization of GUS activity in T1 seedlings for the different Promoter:GUS fusions (e). Red size bars equal 1 mm

We analyzed the expression pattern of *ASR* genes using *GUS* reporter genes driven by their promoter regions. We generated stable transformants harboring these reporters in the M1×R27 hybrid background. The plants positive for *GUS* expression by PCR analysis were further analyzed for GUS activity. pASR1^inf^ and pASR2^inf^ drive GUS activity in several different tissues of young buds: petals, stigma, ovary, and anther filaments (Figure 6a,b). Instead, pASR3^inf^ showed only weak expression in stigma, ovary, and anther filaments (Figure 6c). The expression pattern of pASR1^M1^ is similar to that of pASR1^inf^ and pASR2^inf^, although the *P. inflata* alleles encode inactive proteins (Figure 6d). Mature leaves, stems, and sepals did not display GUS staining in any of the transgenic lines. T1 seedlings of pASR1^inf^:GUS, pASR2^inf^:GUS, and pASR1^M1^:GUS plants germinated on MS medium supplied with antibiotics for 15 d showed strong staining in several organs, while in pASR3^inf^:GUS seedlings grown under the same conditions, no GUS activity was detected (Figure 6e).

We confirmed the expression of each *ASR g*ene in different tissues by RT‐PCR in *P. inflata*, and in the hybrids M1×R27 and M1×V30. The results were consistent with the promoter activity analysis. *ASR1* and *ASR2* were expressed throughout the reproductive organs with higher expression at early stages of floral development. In contrast, *ASR3* was only expressed in stamens and pistils. Furthermore, we found that *ASR1* and *ASR2* were also expressed in leaves and stems (Supporting Information Figure S4a). Interestingly, after the exposure of the plants to intense light conditions, the total expression levels of *ASRs* was induced in vegetative tissues (Supporting Information Figure S4b), which is similar to what had been previously reported for *DPL* and *PHZ* (Albert et al., 2011, 2014). These results further support a possible common origin of *ASR* MYBs and *DPL* and *PHZ* from relatively recent duplication events.

## 4 DISCUSSION

The understanding of how gene expression patterns are generated and how they diverged during evolution is an important question in both developmental and evolution biology. Pigmentation genes provide an attractive model for such studies as their underlying genetic machineries are relatively simple compared to genetic networks regulating body shape and architecture and because changes in pigmentation are easy to score visually (Dembeck, Huang, Carbone, & Mackay, 2015; Gompel et al., 2005; Hoekstra, 2006). In plants, a good description of the pathway for the production of flavonoid pigments makes the use of this system even more attractive (Jaakola, 2013; Koes et al., 2005).

R2R3‐MYBs play in plants a central role in the regulation of the spatiotemporal pattern of expression of structural genes involved in the synthesis and accumulation of pigments, such as anthocyanins (Quattrocchio et al., 1999), condensed tannins (Baudry et al., 2004), betalains (Hatlestad et al., 2015), and the copigments flavonols (Sheehan et al., 2016), and are therefore main players in determining pigmentation patterns. A group of SG6 R2R3‐MYBs has been identified as regulators of the biosynthesis of anthocyanins in different plant parts in all plant species. In this report, we described a newly identified cluster of SG6 MYB genes in *P. inflata*,* ASR1*‐*ASR2*‐*ASR3*. These genes complete the picture of the SG6 phylogenic clade of anthocyanin regulators in this species, together with the already described *AN4*‐*DPL*‐*PHZ* cluster and the *AN2* gene in petunia. The MYB proteins encoded by these genes are all very similar and all belong to the SG6 clade, which is clearly distinct from the clades containing MYBs involved in other aspects of pigmentation, such as vacuolar acidification (PhPH4) (Quattrocchio et al., 2006) and tannin accumulation (AtTT2) (Nesi, Jond, Debeaujon, Caboche, & Lepiniec, 2001) (Figure 2c).

The set of SG6 MYB genes in petunia consists of several members that probably originated by repeated events of duplication that were accompanied by heavy rearrangements of the genomic regions containing the different copies. Such events resulted in the expansion of this MYB group and in the diversification of their promoter regions. Apparently, the *ASR* genes, similar to other anthocyanin MYBs, reside in a very variable region of the genome that is subject to frequent rearrangements (Supplemental note in Bombarely et al., 2016). The amplification of the SG6 MYB family in petunia has occurred after the separation of the *Solanum* and *Petunia* genus, resulting in a petunia‐specific set of anthocyanin MYBs. The synteny of the genomic regions containing these SG6 genes in two wild petunia accessions, *P. axillaris* and *P. inflata*, revealed massive rearrangements that occurred in the time since the separation of these two evolutionary very closely related species, estimated at 0.9 Myr ago (Bombarely et al., 2016). It is conceivable that after the first duplications, and the consequent generation of repeats with highly similar sequences, duplication and rearrangement events happened relatively easily and frequently, due to (local) chromosome miss‐pairing. It is also likely that such events have been under strong positive selection when new flower color patterns generated new (successful) pollination syndromes in (for instance) *Petunia*.

In *P. axillaris* and *P. inflata*, the pattern of deposition of anthocyanins reflects, as expected, the function of the SG6 MYB regulators. In *P. axillaris*,* AN2* is interrupted by a frame shift mutation, the whole *ASR* cluster is lost and *AN4* is not expressed. As a consequence, *P. axillaris* can only synthesize anthocyanins in specific cells of the petal tube, but not in the petal limb and anthers (Supplemental note in Bombarely et al., 2016). In contrast, the *P. inflata* genome contains active copies of *AN2*,* AN4*,* DPL,* and *PHZ*, while *ASR1* and *ASR2* harbor inactivating mutations. The flowers of *P. inflata* have pigmented petals and colored anthers, while the flower pedicels do not show any anthocyanin accumulation. This finding is in agreement with the idea that the variation in expression pattern of SG6 MYB regulators drives the formation of anthocyanin pigmentation patterns.

The *Petunia hybrida* lines originated from manual crosses between different wild petunia species, which lead to the mixing and sorting of the different genomes. *P. hybrida* lines of the Amsterdam petunia collection analyzed in this work contain none, one or two *ASR* clusters. Rearrangement of the *ASR2* promoter region and new insertions in the second intron of the M1 allele of this gene suggests that the dynamic nature of this genomic region born in wild petunias is conserved in the *P. hybrida* lines and possibly resulted in rearrangements even in the short time since people started breeding these species. However, it is also still possible that other (to us unknown) genomes participated in the breeding of *Petunia hybrida* and that the M1 allele originated from one of those. In fact, the wild petunia species *P. violacea* probably contains two copies of the *ASR* cluster, one of which contains *ASR* genes similar to the alleles found in the *P. hybrida* line M1 and the other genes containing the same mutations found in the *P. inflata ASR* cluster. This again supports the idea that patterns of coloration in petunia have been generated by repeated gain (and maybe loss) of new regulators occurring by duplications and differentiation of MYB genes, as well as by repeated loss of function for some of these copies. The differences in promoter regions, the expression patterns of single SG6 MYB genes in petunia, and the effect of mutation and overexpression reported here and in previous studies have shown that these genes differentiated substantially from each other. They can confer pigmentation to different tissues and under different conditions and result in the activation of a partially different set of target genes in the anthocyanin pathway.

The participation of MYBs in the WMBW complex regulating anthocyanin synthesis has been previously shown (Quattrocchio et al., 2006), and in this study, we assessed that this holds also for the three new petunia members of this group of regulators. The display of pigments by plant cells involves, next to the synthesis of anthocyanin molecules, other processes that determine the color of the tissue. One of these is the hyperacidification of the vacuolar lumen that is regulated by a WMBW complex containing a different R2R3‐MYB, encoded in petunia by the *PH4* gene (Quattrocchio et al., 2006), and inducing transcription of the heterodimeric proton pump encoded by the *PH1* and *PH5* genes (Faraco et al., 2014). Similarly, the accumulation of other pigments and copigments that contribute to the coloration of plant tissues is regulated by yet other R2R3‐MYBs, for example, TT2 for tannins (Baudry et al., 2004), MYB‐FL for flavonols (Sheehan et al., 2016), and Y for betalaines (Hatlestad et al., 2015).

All of these MYBs are closely related to each other but differentiated in (a) their function, as they activate distinct sets of target genes, and (b) their expression patterns. The differences in sets of target genes for different MYB members of the WMBW complex are probably not merely due to the evolution of the promoters of the target genes. The acquired specificity is supported by the observation that MYBs of the SG6 clade cannot activate the PH4 targets *PH1* and *PH5*. MYBs of these different clades (e.g., SG5 and SG20) can take part in the WMBW complex, conferring on it large flexibility in target gene specificity and giving rise to a never‐ending variation in pigmentation patterns via the activation of genes involved in the synthesis of different pigments and copigments and acidification of the vacuolar lumen where the pigments are accumulated (ref. PH4 and ref. TT2).

Thus, the characterization of the new R2R3‐MYB cluster containing the genes *ASR1*,* ASR2*, and *ASR3* completes the picture of the petunia MYB genes involved in processes related to the differentiation of petal epidermal cells. These three genes can participate in the formation of WMBW complexes, probably playing a role in the acquisition of pigmentation in very young buds and in other plant parts when these are exposed to very high light. The presence in all plants of numerous genes encoding similar MYB proteins associated with strong rearrangement activity of the genomic region around them provides a genetic basis for the generation of new patterns of pigmentation and might have contributed to the differentiation of species within the *Petunia* genus by affecting their pollination syndromes.

## ACCESSION NUMBERS

Sequence data from this paper have been deposited in GenBank (Supporting Information Table S3).

## AUTHORS’ CONTRIBUTIONS

H.Z., R.K., and F.M.Q. designed the experiments and wrote the manuscript, H. S., L.W., Y.L (Yanbang Li), and J.G. completed gene identification and sequence analysis, X.D. and H. W. cared for the plants, J.Z. and V. P. performed DNA and RNA experiments, H. J. and Z.F. performed RT‐PCR analysis, Y.L (Yanmin Li) generated the transgenic petunia.
