# Supplementary material for: Identification and functional analysis of three new anthocyanin R2R3‐MYB genes in Petunia
Source: Plant Direct. 2019 Jan 21;3(1):e00114. doi: 10.1002/pld3.114 (PMC6508765; doi:10.1002/pld3.114)
Supplement: Supplementary file 1 [file PLD3-3-e00114-s001.docx]

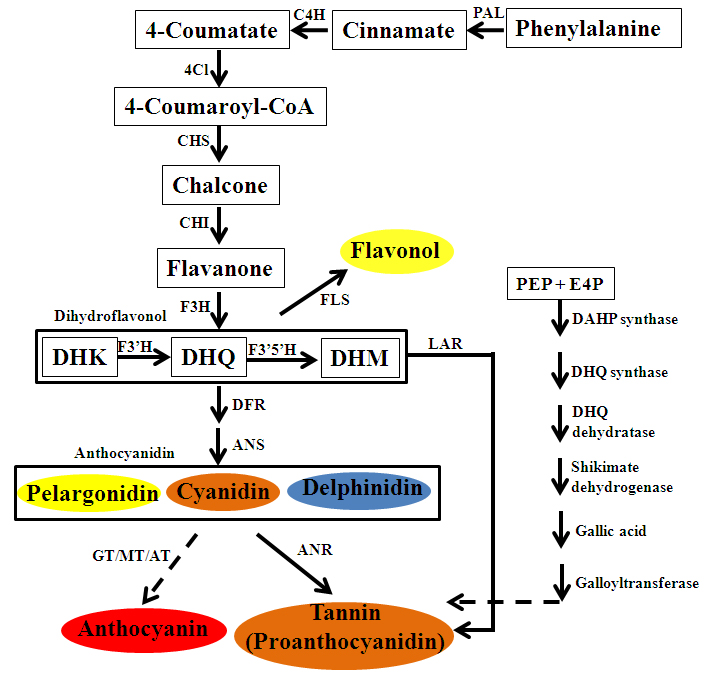


**Supporting Information Figure S1** Anthocyanin and tannin biosynthetic pathway in plants.


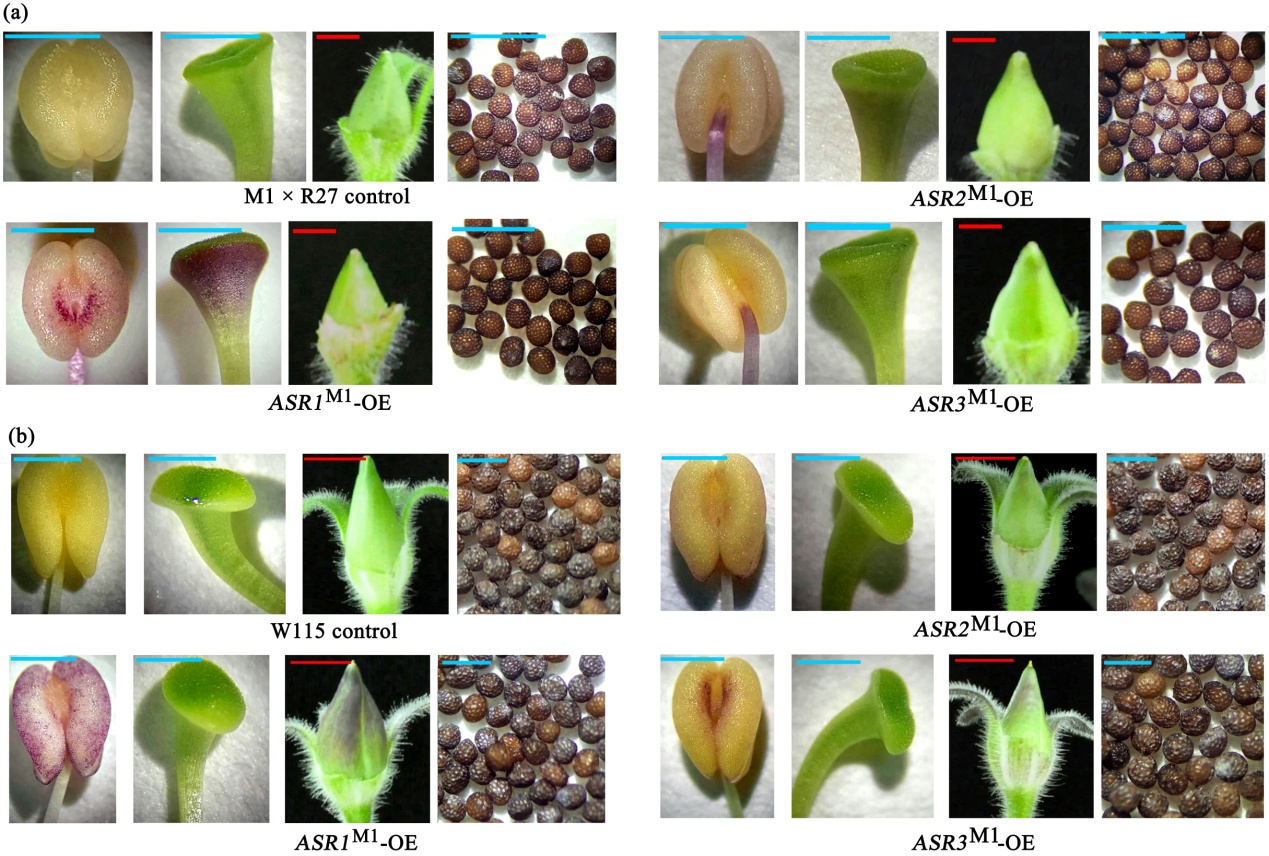


**Supporting Information Figure S2** Floral organs’ phenotype (anthers, pistils, seed pods and seeds) of *ASRs*^M1^ overexpression in M1×R27 (a) and W115 (b). Red size bars equal 5mm and blue size bars 1mm.


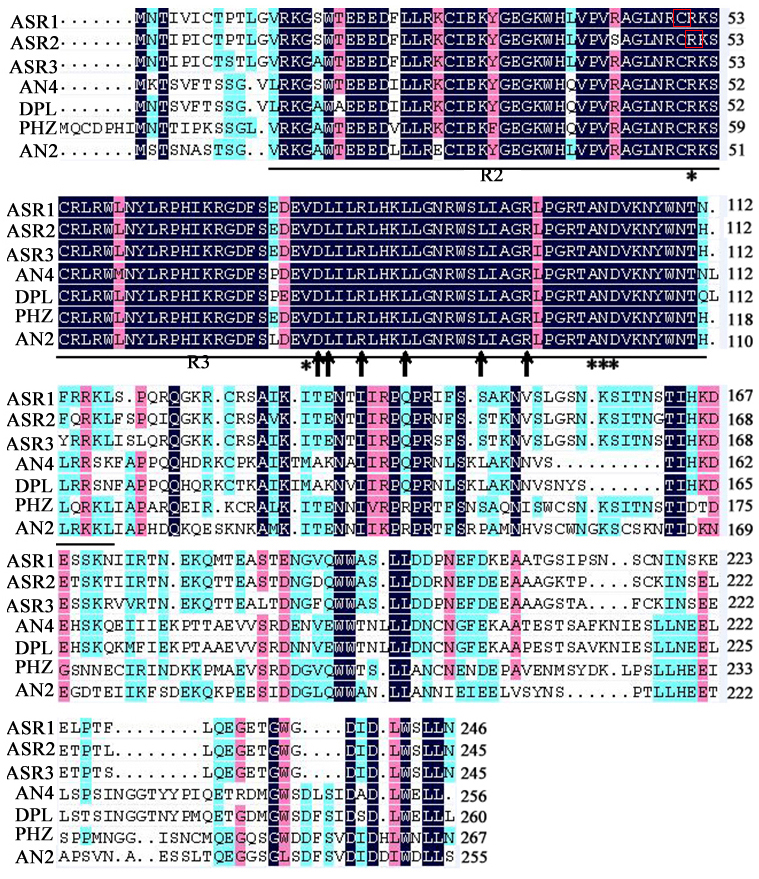


**Supporting Information Figure S3** Multiple alignments of the deduced protein sequences for the *ASR*s and other anthocyanin R2R3-MYB genes from *P. inflata*. Arrowheads indicate the conserved DL_x2_Rx_3_L_x6_L_x3_ motif, which is required for interaction with bHLH transcription factors; and asterisks a conserved R, V, A, and ANDV residues typical of anthocyanin-promoting MYBs (Zimmermann et al., 2004). ASR1 and ASR2 from *P. inflata* were [manual](http://www.baidu.com/link?url=mDc5DyZ5fvBVuKWy00xx10ta2TSSU198pg8xDyBtH8StgJkpen0REbgqYIArxdATNh3neyW4qz3C1N5vPKnrW02x_ozwJ2ctBoy8dQHneRm)ly corrected to restore the reading frame and obtain a full size protein sequence. The red residue marks the position where the mutation is and that was changed in the manually corrected version of the protein.


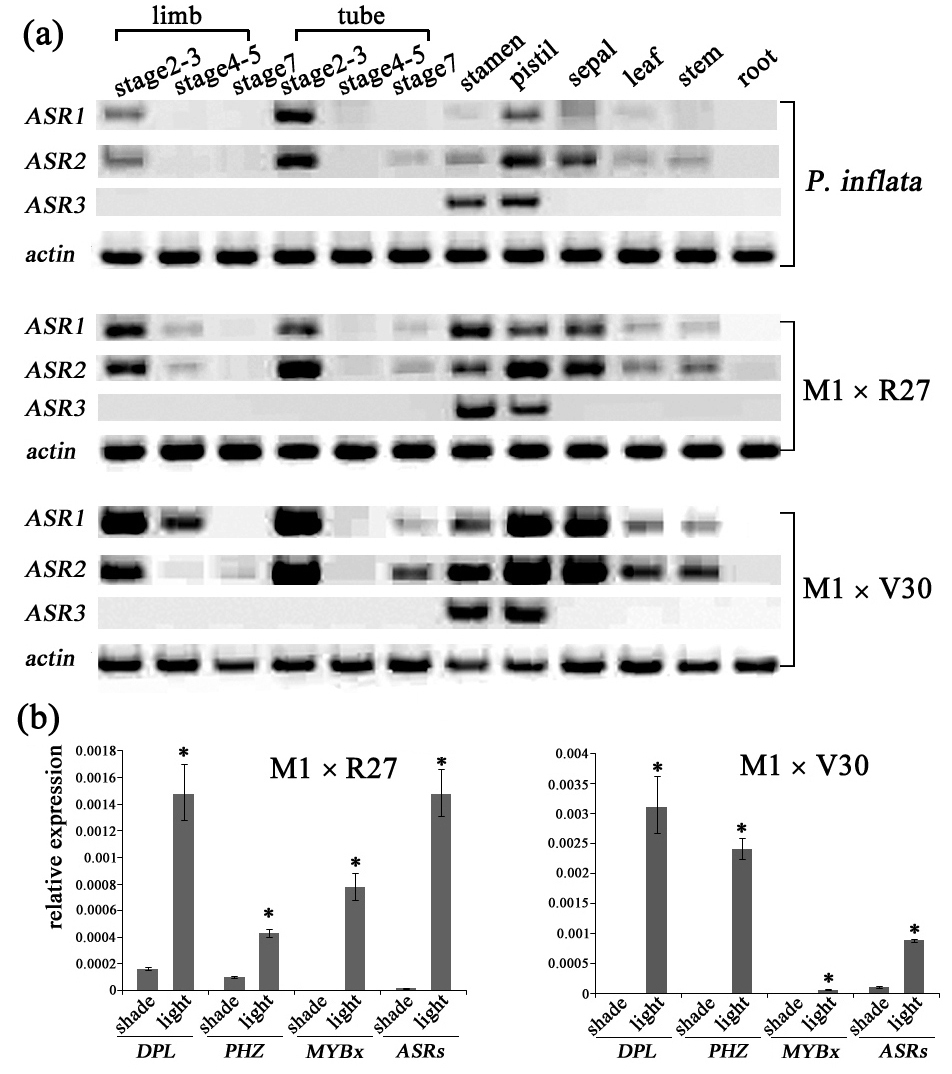


**Supporting Information Figure S4** Expression pattern analysis of anthocyanin genes in *Petunia*. (a)  RT-PCR for *ASR1*, *ASR2* and *ASR3* in different petunia tissues and in different genotypes. (b) Induction of the expression of the anthocyanin regulators *AN4*, *PHZ*, *MYBx*, *ASR1*, *ASR2* and *ASR3* in vegetative tissues by exposure to high intensity light. The qRT-PCR primers we used for *ASR* MYBs (559 and 560) can amplify transcripts from both of *ASR1* and *ASR2* genes; therefore the graph shows the total expression of them.
