# Supplementary material for: Identification and functional analysis of three new anthocyanin R2R3‐MYB genes in Petunia
Source: Plant Direct. 2019 Jan 21;3(1):e00114. doi: 10.1002/pld3.114 (PMC6508765; doi:10.1002/pld3.114)
Supplement: Supplementary file 2 [file PLD3-3-e00114-s002.docx]

## Supporting Information Table S1 *ASR* genes in petunia wild accessions and hybrid lines.

| **Plant name** | **Characteristics of phenotype and origin** | ***ASRs* situation**  **(*ASR*- wild type, *Asr*-mutated and ‘-’ means no *ASR*)** |
| --- | --- | --- |
| *P. inflata* | violet corolla | *Asr* |
| *P. axillaris* | white corolla | -/- |
| *P. exserta* (Bern) | red corolla | -/- |
| *P. parodii* (Michigan) | white corolla | -/- |
| *P. parodii* (Michigan) | white corolla | -/- |
| *P. violacea* (Dijon) | violet corolla | two types (*ASR* and *Asr*) |
| *P. parodii* (Michigan) | white corolla | -/- |
| *P. parodii* (Michigan) | white corolla | -/- |
| *P. violacea* (Dijon) | violet corolla | two types (*ASR* and *Asr)* |
| *P. exserta* (Bern) | red corolla | -/- |
| *P. axillaris* (Kew) | white corolla | -/- |
| *P. axillaris* (Uruguay) | white corolla | -/- |
| *P. axillaris* (Kew) | white corolla | -/- |
| *P. axillaris* (Uruguay) | white | -/- |
| V30 | V113-1 × V113-2, violet corolla | *ASR* |
| R27 | V57-16 × V57-14, red corolla | -/- |
| M1 | V112-1 × V112-2, magenta corolla | *ASR* |
| W115 | white | -/- |
| W225 | T18-1 × T18-2, white corolla | -/- |
| V42 | *an8*-, *ph1* blue, purple, Deep Blauw | -/- |
| W59 | *an2*-, lightly colored, Dijon | *ASR* |
| W170 | *an3*-, white unstable, W138 | -/- |
| V23 | Blue violet, Blauw Zwerg | *ASR* |
| M29 | *Hf*, *mt*, *mf*-, magenta, R4 × V4 | -/- |
| V48 | *dg1*, Blue violet, Straub, klein donkergroen blad | -/- |
| V70 | *ph5*-, Blue violet, (V12 × V62) × V62 | -/- |
| R67 | *ph1*, purple, red, M26 × R3 | *ASR* |
| R153 | *ph6*-, *an1*-, purplish red + red spots, G621-2 | *ASR* |
| M87 | *an1*+ progenitor of *an1*- × 2366, magenta corolla, white/yellow tube (S&G) | -/- |
| M80 | ‘V32 × V55 (M30, R93)’ | -/- |
| W242 | *an1*-, White | *ASR* |
| W82 | *an2*, light purple, Dijon | -/- |
| V69 | *ph5*-, blue violet, (V12 × V62) × S | -/- |
| V64 | *ph4*-, (V32 × V55) × M30 | -/- |
| V63 | *ph4*, (V32 × V55) × M30) × R93 | -/- |
| R175 | R27-like-S2096, red, W138, Bleiswijk | -/- |
| W137 | *an11*-, unstable, white unstable | -/- |
| V26 | *ph2*-, blue dark violet, Blue Jay | *ASR* |
| R143(*ph3*, mutant) | pinkish flower | *ASR* |
| R143(*ph3*, WT) | *ph3*-, purple red, R3 × R122 | *ASR* |

**Supporting Information Table S2** Genes or sequences appearing in the synteny analysis reported in Figure 1.

| **No.** | **Gene/ unknown sequence** | **Accession number** | | | | |
| --- | --- | --- | --- | --- | --- | --- |
|  |  | ***P. inflata*** | ***P. axillaris*** | ***S. lycopersicum*** | ***S. pennellii*** | ***S. tuberosum*** |
| 1 | Ras-related small GTP-binding family protein | [Peinf101Scf00113g06019.1](https://solgenomics.net/tools/blast/show_match_seq.pl?blast_db_id=273;id=Peinf101Scf00113g06019.1;hilite_coords=1-651) | [Peaxi162Scf00407g00632.1](https://solgenomics.net/tools/blast/show_match_seq.pl?blast_db_id=269;id=Peaxi162Scf00407g00632.1;hilite_coords=1-643) | Solyc10g086350 |  |  |
| 2 | ATP binding microtubule motor family protein | [Peinf101Scf00113g06021.1](https://solgenomics.net/tools/blast/show_match_seq.pl?blast_db_id=273;id=Peinf101Scf00113g06021.1;hilite_coords=85-3108) | [Peaxi162Scf00407g00069.1](https://solgenomics.net/tools/blast/show_match_seq.pl?blast_db_id=269;id=Peaxi162Scf00407g00069.1;hilite_coords=73-1001) | Solyc10g086340 |  |  |
| 3 | RPL23AB ribosomal protein L23AB | partial |  | Solyc10g086330 |  |  |
| 4 | PIP5K9 phosphatidyl inositol monophosphate 5-kinase | [Peinf101Scf00113g06018.1](https://solgenomics.net/tools/blast/show_match_seq.pl?blast_db_id=273;id=Peinf101Scf00113g06018.1;hilite_coords=1-1578) | [Peaxi162Scf00435g01211.1](https://solgenomics.net/tools/blast/show_match_seq.pl?blast_db_id=269;id=Peaxi162Scf00435g01211.1;hilite_coords=153-1578) | Solyc10g086320 | [Sopen10g035700](https://solgenomics.net/tools/blast/show_match_seq.pl?blast_db_id=261;id=Sopen10g035700;hilite_coords=1-1581) | [Sotub10g028520.1.1](https://solgenomics.net/tools/blast/show_match_seq.pl?blast_db_id=208;id=Sotub10g028520.1.1;hilite_coords=1-2367) |
| 5 | Unknown protein | Transposon |  | Solyc10g086230 | [Sopen10g035610](https://solgenomics.net/tools/blast/show_match_seq.pl?blast_db_id=261;id=Sopen10g035610;hilite_coords=1-243) |  |
| 6 | Uncharacterized protein (GTP-binding) | [Peinf101Scf00113g05008.1](https://solgenomics.net/tools/blast/show_match_seq.pl?blast_db_id=273;id=Peinf101Scf00113g05008.1;hilite_coords=24-600) | [Peaxi162Scf00658g00009.1](https://solgenomics.net/tools/blast/show_match_seq.pl?blast_db_id=269;id=Peaxi162Scf00658g00009.1;hilite_coords=25-597) | Solyc10g086310 | [Sopen10g035700](https://solgenomics.net/tools/blast/show_match_seq.pl?blast_db_id=261;id=Sopen10g035700;hilite_coords=2551-3178) |  |
| 7 | Transposon |  |  |  |  |  |
| 8 | MAT3 (Methionine Adenosyltransferase3) | [Peinf101Scf00113g01001.1](https://solgenomics.net/tools/blast/show_match_seq.pl?blast_db_id=273;id=Peinf101Scf00113g01001.1;hilite_coords=1-1173) | [Peaxi162Scf00578g00014.1](https://solgenomics.net/tools/blast/show_match_seq.pl?blast_db_id=269;id=Peaxi162Scf00578g00014.1;hilite_coords=1-1173) | [Solyc10g083970.1](https://solgenomics.net/tools/blast/show_match_seq.pl?blast_db_id=297;id=Solyc10g083970.1;hilite_coords=1-1173) | [Sopen10g033420](https://solgenomics.net/tools/blast/show_match_seq.pl?blast_db_id=261;id=Sopen10g033420;hilite_coords=1-1173) | [Sotub10g025090.1.1](https://solgenomics.net/tools/blast/show_match_seq.pl?blast_db_id=208;id=Sotub10g025090.1.1;hilite_coords=1-1173) |
| 9 | Big genomic sequences | >20000bp | >20000bp |  |  |  |
| 10 | Heavy metal associated protein | [Peinf101Scf00622g09005.1](https://solgenomics.net/tools/blast/show_match_seq.pl?blast_db_id=273;id=Peinf101Scf00622g09005.1;hilite_coords=4-270) | [Peaxi162Scf00488g00011.1](https://solgenomics.net/tools/blast/show_match_seq.pl?blast_db_id=269;id=Peaxi162Scf00488g00011.1;hilite_coords=172-402) | Solyc10g086280 | [Sopen10g035670](https://solgenomics.net/tools/blast/show_match_seq.pl?blast_db_id=261;id=Sopen10g035670;hilite_coords=136-264) | [ST4.03ch10](https://solgenomics.net/tools/blast/show_match_seq.pl?blast_db_id=263;id=ST4.03ch10;hilite_coords=51611633-51611780)(51611302-51628288) |
| 11 | Unknown protein | [Peinf101Scf02985g01015.1](https://solgenomics.net/tools/blast/show_match_seq.pl?blast_db_id=273;id=Peinf101Scf02985g01015.1;hilite_coords=522-712) |  |  |  |  |
| 12 | DNA polymerase alpha subunit B | [Peinf101Scf00622g09009.1](https://solgenomics.net/tools/blast/show_match_seq.pl?blast_db_id=273;id=Peinf101Scf00622g09009.1;hilite_coords=109-283) |  |  |  |  |
| 13 | Transposon |  |  |  |  |  |
| 14 | Transposon |  |  |  |  |  |
| 15 | UDP-Glycosyltranferase superfamily protein | [Peinf101Scf00622g05007.1](https://solgenomics.net/tools/blast/show_match_seq.pl?blast_db_id=273;id=Peinf101Scf00622g05007.1;hilite_coords=529-1446) | [Peaxi162Scf00488g00026.1](https://solgenomics.net/tools/blast/show_match_seq.pl?blast_db_id=269;id=Peaxi162Scf00488g00026.1;hilite_coords=529-1431) |  |  |  |
| 16 | Ycf2 protein | [Peinf101Scf00622g05003.1](https://solgenomics.net/tools/blast/show_match_seq.pl?blast_db_id=273;id=Peinf101Scf00622g05003.1;hilite_coords=1-548) | [Peaxi162Scf00040g00123.1](https://solgenomics.net/tools/blast/show_match_seq.pl?blast_db_id=269;id=Peaxi162Scf00040g00123.1;hilite_coords=931-1478) | S[olyc08g029400](https://solgenomics.net/tools/blast/show_match_seq.pl?blast_db_id=297;id=Solyc08g029400.2;hilite_coords=41-536) | [Sopen02g009120](https://solgenomics.net/tools/blast/show_match_seq.pl?blast_db_id=261;id=Sopen02g009120;hilite_coords=1822-2369) | [Sotub12g020050.1.1](https://solgenomics.net/tools/blast/show_match_seq.pl?blast_db_id=208;id=Sotub12g020050.1.1;hilite_coords=220-726) |
| 17 | Big genomic sequences | >20000bp | >20000bp |  |  |  |
| 18 | ATOPR2 12-oxophytodienoate reductase 2 | [Peinf101Scf00665g22053.1](https://solgenomics.net/tools/blast/show_match_seq.pl?blast_db_id=273;id=Peinf101Scf00665g22053.1;hilite_coords=60-1081) | [Peaxi162Scf00688g00361.1](https://solgenomics.net/tools/blast/show_match_seq.pl?blast_db_id=269;id=Peaxi162Scf00688g00361.1;hilite_coords=46-904) | Solyc10g086220 | [Sopen10g035600](https://solgenomics.net/tools/blast/show_match_seq.pl?blast_db_id=261;id=Sopen10g035600;hilite_coords=11-892) | [ST4.03ch10](https://solgenomics.net/tools/blast/show_match_seq.pl?blast_db_id=263;id=ST4.03ch10;hilite_coords=51907847-51908346) (51868487-51870211) |
| 19 | Translin family protein | [Peinf101Scf00622g02009.1](https://solgenomics.net/tools/blast/show_match_seq.pl?blast_db_id=273;id=Peinf101Scf00622g02009.1;hilite_coords=419-855) | [Peaxi162Scf00488g00042.1](https://solgenomics.net/tools/blast/show_match_seq.pl?blast_db_id=269;id=Peaxi162Scf00488g00042.1;hilite_coords=63-717) | Solyc10g086210 | [Sopen10g035590](https://solgenomics.net/tools/blast/show_match_seq.pl?blast_db_id=261;id=Sopen10g035590;hilite_coords=1-882) | [ST4.03ch10](https://solgenomics.net/tools/blast/show_match_seq.pl?blast_db_id=263;id=ST4.03ch10;hilite_coords=51907847-51908346) (51903871-51908130) |
| 20 | RNA-dependent DNA polymerase |  |  |  | Sopen10g035580 |  |
| 21 | 125 kDa kinesin-related protein | [Peinf101Scf00113g06021.1](https://solgenomics.net/tools/blast/show_match_seq.pl?blast_db_id=273;id=Peinf101Scf00113g06021.1;hilite_coords=429-641) | [Peaxi162Scf00407g00069.1](https://solgenomics.net/tools/blast/show_match_seq.pl?blast_db_id=269;id=Peaxi162Scf00407g00069.1;hilite_coords=429-629) | [Solyc10g086335](https://solgenomics.net/tools/blast/show_match_seq.pl?blast_db_id=297;id=Solyc10g086335.1;hilite_coords=464-697) | [Sopen10g035720](https://solgenomics.net/tools/blast/show_match_seq.pl?blast_db_id=261;id=Sopen10g035720;hilite_coords=563-796) | [Sotub10g028500.1.1](https://solgenomics.net/tools/blast/show_match_seq.pl?blast_db_id=208;id=Sotub10g028500.1.1;hilite_coords=464-697) |
| 22 | GAI-like protein 1 |  |  | [Solyc10g086380.1](https://solgenomics.net/tools/blast/show_match_seq.pl?blast_db_id=297;id=Solyc10g086380.1;hilite_coords=389-1542) | [Sopen10g035750](https://solgenomics.net/tools/blast/show_match_seq.pl?blast_db_id=261;id=Sopen10g035750;hilite_coords=605-1268) | [Sotub10g028470.1.1](https://solgenomics.net/tools/blast/show_match_seq.pl?blast_db_id=208;id=Sotub10g028470.1.1;hilite_coords=1-1533) |
| 23 | BRI1-KD interacting protein 130 | Peinf101Scf00113g07018.1 | [Peaxi162Scf00407g00067.1](https://solgenomics.net/tools/blast/show_match_seq.pl?blast_db_id=269;id=Peaxi162Scf00407g00067.1;hilite_coords=167-729) | Solyc10g086360 | [Sopen10g035740](https://solgenomics.net/tools/blast/show_match_seq.pl?blast_db_id=261;id=Sopen10g035740;hilite_coords=1-735) | [Sotub10g028480.1.1](https://solgenomics.net/tools/blast/show_match_seq.pl?blast_db_id=208;id=Sotub10g028480.1.1;hilite_coords=304-735) |
| 24 | Adenosine kinase 2 | [Peinf101Scf00622g01002.1](https://solgenomics.net/tools/blast/show_match_seq.pl?blast_db_id=273;id=Peinf101Scf00622g01002.1;hilite_coords=698-884) |  | [Solyc10g086190.2](https://solgenomics.net/tools/blast/show_match_seq.pl?blast_db_id=297;id=Solyc10g086190.2;hilite_coords=160-1044) | [Sopen10g035570](https://solgenomics.net/tools/blast/show_match_seq.pl?blast_db_id=261;id=Sopen10g035570;hilite_coords=101-1017) | [Sotub09g007480.1.1](https://solgenomics.net/tools/blast/show_match_seq.pl?blast_db_id=208;id=Sotub09g007480.1.1;hilite_coords=101-1019) |
| 25 | Serine protease family | [Peinf101Scf02633g04014.1](https://solgenomics.net/tools/blast/show_match_seq.pl?blast_db_id=273;id=Peinf101Scf02633g04014.1;hilite_coords=1630-3567) | [Peaxi162Scf00118g00223.1](https://solgenomics.net/tools/blast/show_match_seq.pl?blast_db_id=269;id=Peaxi162Scf00118g00223.1;hilite_coords=160-1855) | Solyc03g043660 | [Sopen03g007520](https://solgenomics.net/tools/blast/show_match_seq.pl?blast_db_id=261;id=Sopen03g007520;hilite_coords=1-3306) | [Sotub02g029950.1.1](https://solgenomics.net/tools/blast/show_match_seq.pl?blast_db_id=208;id=Sotub02g029950.1.1;hilite_coords=1867-3336) |
| 26 | ARMADILLO/BETA-CATENIN repeat family protein | [Peinf101Scf00468g01018.1](https://solgenomics.net/tools/blast/show_match_seq.pl?blast_db_id=273;id=Peinf101Scf00468g01018.1;hilite_coords=73-1146) | [Peaxi162Scf00118g00222.1](https://solgenomics.net/tools/blast/show_match_seq.pl?blast_db_id=271;id=Peaxi162Scf00118g00222.1;hilite_coords=3-343) | Solyc03g043700 | [Sopen03g007540](https://solgenomics.net/tools/blast/show_match_seq.pl?blast_db_id=261;id=Sopen03g007540;hilite_coords=1-1083) | [Sotub02g011050.1.1](https://solgenomics.net/tools/blast/show_match_seq.pl?blast_db_id=208;id=Sotub02g011050.1.1;hilite_coords=67-1116) |
| 27 | Serine/threonine protein kinase | [Peinf101Scf00691g00002.1](https://solgenomics.net/tools/blast/show_match_seq.pl?blast_db_id=273;id=Peinf101Scf00691g00002.1;hilite_coords=71-1961) | [Peaxi162Scf00118g00063.1](https://solgenomics.net/tools/blast/show_match_seq.pl?blast_db_id=269;id=Peaxi162Scf00118g00063.1;hilite_coords=71-1961) | Solyc03g043710 | [Sopen03g007550](https://solgenomics.net/tools/blast/show_match_seq.pl?blast_db_id=261;id=Sopen03g007550;hilite_coords=10-1953) | [ST4.03ch03](https://solgenomics.net/tools/blast/show_match_seq.pl?blast_db_id=263;id=ST4.03ch03;hilite_coords=5635958-5637897) (5635958-5637899) |
| 28 | Trimeric coiled-coil oligomerisation domain of matrilin |  |  | Solyc03g043720 | [Sopen03g007570](https://solgenomics.net/tools/blast/show_match_seq.pl?blast_db_id=261;id=Sopen03g007570;hilite_coords=1-258) | [ST4.03ch03](https://solgenomics.net/tools/blast/show_match_seq.pl?blast_db_id=263;id=ST4.03ch03;hilite_coords=5657321-5657689) (5657000-5657700) |

**Supporting Information Table S3** Accession numbers of genes or proteins appearing in this paper.

| **Name** | **Genome location** | **Origin Plant** | **Accession number in NCBI**  **or ID number in genome** |
| --- | --- | --- | --- |
| ASR1^inf^ | [Peinf101Scf00622](https://solgenomics.net/tools/blast/show_match_seq.pl?blast_db_id=276;id=Peinf101Scf00622;hilite_coords=709755-711265)(709755..711265) | *P. inflata* | MF682093 |
| ASR2^inf^ | [Peinf101Scf00622](https://solgenomics.net/tools/blast/show_match_seq.pl?blast_db_id=276;id=Peinf101Scf00622;hilite_coords=709755-711265)(791412..792946) | *P. inflata* | MF682097 |
| ASR3^inf^ | [Peinf101Scf00622](https://solgenomics.net/tools/blast/show_match_seq.pl?blast_db_id=276;id=Peinf101Scf00622;hilite_coords=709755-711265)(898092..899594) | *P. inflata* | MF623311 |
|  |  |  |  |
| ASR1^M1^ |  | *P. hybrid* M1 | MF623315 |
| ASR2^M1^ |  | *P. hybrid* M1 | MF623312 |
| ASR3^M1^ |  | *P. hybrid* M1 | MF623309 |
| ASR1^V30^ |  | *P. hybrid* V30 | MF623314 |
| ASR2^V30^ |  | *P. hybrid* V30 | MF623313 |
| ASR3^V30^ |  | *P. hybrid* V30 | MF623310 |
| SlAN2a | [SL3.0ch10](https://solgenomics.net/tools/blast/show_match_seq.pl?blast_db_id=281;id=SL3.0ch10;hilite_coords=65299527-65300524)(69256490..65258047) | *S. lycopersicum* | [Solyc10g086260.2](https://solgenomics.net/tools/blast/show_match_seq.pl?blast_db_id=297;id=Solyc10g086260.2;hilite_coords=133-248) |
| SlAN2b | [SL3.0ch10](https://solgenomics.net/tools/blast/show_match_seq.pl?blast_db_id=281;id=SL3.0ch10;hilite_coords=65299527-65300524)(69260733..65261729) | *S. lycopersicum* | [Solyc10g086250.2](https://solgenomics.net/tools/blast/show_match_seq.pl?blast_db_id=297;id=Solyc10g086250.2;hilite_coords=258-828) |
| SlAN2c | [SL3.0ch10](https://solgenomics.net/tools/blast/show_match_seq.pl?blast_db_id=281;id=SL3.0ch10;hilite_coords=65299527-65300524)(69267742..65268676) | *S. lycopersicum* | [Solyc10g086270.2](https://solgenomics.net/tools/blast/show_match_seq.pl?blast_db_id=297;id=Solyc10g086270.2;hilite_coords=259-780) |
| SlAN2d | [SL3.0ch10](https://solgenomics.net/tools/blast/show_match_seq.pl?blast_db_id=281;id=SL3.0ch10;hilite_coords=65299527-65300524)(69290414..65291769) | *S. lycopersicum* | [Solyc10g086290.2](https://solgenomics.net/tools/blast/show_match_seq.pl?blast_db_id=297;id=Solyc10g086290.2;hilite_coords=318-861) |
| SlAN2e | [SL3.0ch10](https://solgenomics.net/tools/blast/show_match_seq.pl?blast_db_id=281;id=SL3.0ch10;hilite_coords=65299527-65300524)(69299527..65300524) | *S. lycopersicum* | [Solyc10g086290.2](https://solgenomics.net/tools/blast/show_match_seq.pl?blast_db_id=297;id=Solyc10g086290.2;hilite_coords=533-820) |
| SpAN2a | [Spenn-ch10](https://solgenomics.net/tools/blast/show_match_seq.pl?blast_db_id=226;id=Spenn-ch10;hilite_coords=82156236-82157219)(82115925..82117052) | *S. pennellii* | [Sopen10g035640](https://solgenomics.net/tools/blast/show_match_seq.pl?blast_db_id=261;id=Sopen10g035640;hilite_coords=245-810) |
| SpAN2b | [Spenn-ch10](https://solgenomics.net/tools/blast/show_match_seq.pl?blast_db_id=226;id=Spenn-ch10;hilite_coords=82156236-82157219)(82119734..821170636) | *S. pennellii* | [Sopen10g035650](https://solgenomics.net/tools/blast/show_match_seq.pl?blast_db_id=261;id=Sopen10g035650;hilite_coords=232-402) |
| SpAN2c | [Spenn-ch10](https://solgenomics.net/tools/blast/show_match_seq.pl?blast_db_id=226;id=Spenn-ch10;hilite_coords=82156236-82157219)(82128447..82127555) | *S. pennellii* | [Sopen10g035660](https://solgenomics.net/tools/blast/show_match_seq.pl?blast_db_id=261;id=Sopen10g035660;hilite_coords=259-740) |
| SpAN2d | [Spenn-ch10](https://solgenomics.net/tools/blast/show_match_seq.pl?blast_db_id=226;id=Spenn-ch10;hilite_coords=82156236-82157219)(82149308..82150662) | *S. pennellii* | [Sopen10g035680](https://solgenomics.net/tools/blast/show_match_seq.pl?blast_db_id=261;id=Sopen10g035680;hilite_coords=252-801) |
| SpAN2e | [Spenn-ch10](https://solgenomics.net/tools/blast/show_match_seq.pl?blast_db_id=226;id=Spenn-ch10;hilite_coords=82156236-82157219)(82156236..82157219) | *S. pennellii* | [Sopen10g035690](https://solgenomics.net/tools/blast/show_match_seq.pl?blast_db_id=261;id=Sopen10g035690;hilite_coords=1-312) |
| StAN2a | [ST4.03ch10](https://solgenomics.net/tools/blast/show_match_seq.pl?blast_db_id=263;id=ST4.03ch10;hilite_coords=51749218-51750359)(51749218..51750359) | *S. tuberosum* | [PGSC0003DMC400024606](https://solgenomics.net/tools/blast/show_match_seq.pl?blast_db_id=186;id=PGSC0003DMC400024606;hilite_coords=117-627) |
| StAN2b | [ST4.03ch10](https://solgenomics.net/tools/blast/show_match_seq.pl?blast_db_id=263;id=ST4.03ch10;hilite_coords=51749218-51750359)(51725194..51726374) | *S. tuberosum* | [PGSC0003DMC400024608](https://solgenomics.net/tools/blast/show_match_seq.pl?blast_db_id=186;id=PGSC0003DMC400024608;hilite_coords=76-639) |
| StAN2c | [ST4.03ch10](https://solgenomics.net/tools/blast/show_match_seq.pl?blast_db_id=263;id=ST4.03ch10;hilite_coords=51749218-51750359)(51667537..51668474) | *S. tuberosum* |  |
| StAN2d | [ST4.03ch10](https://solgenomics.net/tools/blast/show_match_seq.pl?blast_db_id=263;id=ST4.03ch10;hilite_coords=51749218-51750359)(51469785..51471461) | *S. tuberosum* | [Sotub10g028550.1.1](https://solgenomics.net/tools/blast/show_match_seq.pl?blast_db_id=208;id=Sotub10g028550.1.1;hilite_coords=253-798) |
| StAN2e | [ST4.03ch10](https://solgenomics.net/tools/blast/show_match_seq.pl?blast_db_id=263;id=ST4.03ch10;hilite_coords=51749218-51750359)(51457499..51458492) | *S. tuberosum* | [Sotub10g028540.1.1](https://solgenomics.net/tools/blast/show_match_seq.pl?blast_db_id=208;id=Sotub10g028540.1.1;hilite_coords=259-747) |
| SlANT1 |  | *S. lycopersicum* | NM_001247488 |
| F3’5’H-1^inf^ | [Peinf101Scf00872](https://solgenomics.net/tools/blast/show_match_seq.pl?blast_db_id=276;id=Peinf101Scf00872;hilite_coords=108846-111623)(108846..111623) | *P. inflata* | MF682096 |
| F3’5’H-1^axi^ | [Peaxi162Scf00150](https://solgenomics.net/tools/blast/show_match_seq.pl?blast_db_id=272;id=Peaxi162Scf00150;hilite_coords=228368-232659)(228368..232659) | *P. axillaris* |  |
| F3’5’H-1^R27^ |  | *P. hybrid* R27 | MF682095 |
| F3’5’H-1^V30^ |  | *P. hybrid* V30 | MF682094 |
| PH4^inf^ | [Peinf101Scf02429](https://solgenomics.net/tools/blast/show_match_seq.pl?blast_db_id=276;id=Peinf101Scf02429;hilite_coords=28119-28971)(27421..28971) | *P. inflata* | [Peinf101Scf02429g00002.1](https://solgenomics.net/tools/blast/show_match_seq.pl?blast_db_id=273;id=Peinf101Scf02429g00002.1;hilite_coords=65-375) |
| PH4^axi^ | [Peaxi162Scf00349](https://solgenomics.net/tools/blast/show_match_seq.pl?blast_db_id=272;id=Peaxi162Scf00349;hilite_coords=564419-565977)(564419..565977) | *P. axillaris* | [Peaxi162Scf00349g00057.1](https://solgenomics.net/tools/blast/show_match_seq.pl?blast_db_id=269;id=Peaxi162Scf00349g00057.1;hilite_coords=295-846) |
| AN2^inf^ | [Peinf101Scf02633](https://solgenomics.net/tools/blast/show_match_seq.pl?blast_db_id=276;id=Peinf101Scf02633;hilite_coords=502558-504269)(502558..504269) | *P. inflata* | [Peinf101Scf02633g05002.1](https://solgenomics.net/tools/blast/show_match_seq.pl?blast_db_id=273;id=Peinf101Scf02633g05002.1;hilite_coords=130-399) |
| AN2^axi^ | [Peaxi162Scf00118](https://solgenomics.net/tools/blast/show_match_seq.pl?blast_db_id=272;id=Peaxi162Scf00118;hilite_coords=356538-358109)(356538..358109) | *P. axillaris* | [Peaxi162Scf00118g00310.1](https://solgenomics.net/tools/blast/show_match_seq.pl?blast_db_id=269;id=Peaxi162Scf00118g00310.1;hilite_coords=1-132) |
| AN4^inf^ | [Peinf101Scf00113](https://solgenomics.net/tools/blast/show_match_seq.pl?blast_db_id=276;id=Peinf101Scf00113;hilite_coords=175827-177062)(175827..177062) | *P. inflata* | [Peinf101Scf00113g01010.1](https://solgenomics.net/tools/blast/show_match_seq.pl?blast_db_id=273;id=Peinf101Scf00113g01010.1;hilite_coords=133-450) |
| AN4I^axi^ | [Peaxi162Scf00578](https://solgenomics.net/tools/blast/show_match_seq.pl?blast_db_id=272;id=Peaxi162Scf00578;hilite_coords=88536-89754)(88536..89754) | *P. axillaris* | [Peaxi162Scf00578g00008.1](https://solgenomics.net/tools/blast/show_match_seq.pl?blast_db_id=269;id=Peaxi162Scf00578g00008.1;hilite_coords=1-135) |
| AN4II^axi^ | [Peaxi162Scf00578](https://solgenomics.net/tools/blast/show_match_seq.pl?blast_db_id=272;id=Peaxi162Scf00578;hilite_coords=88536-89754)(56575..57794) | *P. axillaris* | [Peaxi162Scf00578g00007.1](https://solgenomics.net/tools/blast/show_match_seq.pl?blast_db_id=269;id=Peaxi162Scf00578g00007.1;hilite_coords=166-675) |
| DPL^inf^ | [Peinf101Scf00113](https://solgenomics.net/tools/blast/show_match_seq.pl?blast_db_id=276;id=Peinf101Scf00113;hilite_coords=236820-239727)(236820..239727) | *P. inflata* | [Peinf101Scf00113g02010.1](https://solgenomics.net/tools/blast/show_match_seq.pl?blast_db_id=273;id=Peinf101Scf00113g02010.1;hilite_coords=133-696) |
| DPL^axi^ | [Peaxi162Scf01210](https://solgenomics.net/tools/blast/show_match_seq.pl?blast_db_id=272;id=Peaxi162Scf01210;hilite_coords=28499-29800)(28499..29800) | *P. axillaris* | [Peaxi162Scf01210g00002.1](https://solgenomics.net/tools/blast/show_match_seq.pl?blast_db_id=269;id=Peaxi162Scf01210g00002.1;hilite_coords=166-693) |
| PHZ^axi^ | [Peaxi162Scf00658](https://solgenomics.net/tools/blast/show_match_seq.pl?blast_db_id=272;id=Peaxi162Scf00658;hilite_coords=91128-93115)(91128..93115) | *P. axillaris* | [Peaxi162Scf00658g00110.1](https://solgenomics.net/tools/blast/show_match_seq.pl?blast_db_id=269;id=Peaxi162Scf00658g00110.1;hilite_coords=283-726) |
| PHZ^inf^ | [Peinf101Scf00113](https://solgenomics.net/tools/blast/show_match_seq.pl?blast_db_id=276;id=Peinf101Scf00113;hilite_coords=175827-177062)(433388..435360) | *P. inflata* | [Peinf101Scf00113g04004.1](https://solgenomics.net/tools/blast/show_match_seq.pl?blast_db_id=273;id=Peinf101Scf00113g04004.1;hilite_coords=148-519) |
| MYB-FL | Peinf101Scf01050:439139..445824 | *P. inflata* | [Peinf101Scf01050g04008.1](https://solgenomics.net/tools/blast/show_match_seq.pl?blast_db_id=273;id=Peinf101Scf01050g04008.1;hilite_coords=1-963) |
| MYB-FL | Peaxi162Scf00886:209001..211100 | *P. axillaris* | [Peaxi162Scf00886g00028.1](https://solgenomics.net/tools/blast/show_match_seq.pl?blast_db_id=269;id=Peaxi162Scf00886g00028.1;hilite_coords=1-1041) |
| PH4 | Peinf101Scf02429:27110..29280 | *P. inflata* | Peinf101Scf02429g00002.1 |
| TT2-like | Peinf101Scf00968:142142..143956 | *P. inflata* | [Peinf101Scf00968g01003.1](https://solgenomics.net/tools/blast/show_match_seq.pl?blast_db_id=273;id=Peinf101Scf00968g01003.1;hilite_coords=166-654) |
| AtMYB75/PAP1 | Chr01:21233555..21235292 | *Arabidopsis* | AT1G56650.1 |
| AtMYB90/PAP2 | Chr01:4753099..4755656 | *Arabidopsis* | AT1G66390.1 |
| AtMYB113 | Chr01:24753589..24754761 | *Arabidopsis* | AT1G66370.1 |
| AtMYB114 | Chr01:24757298..24758935 | *Arabidopsis* | AT1G66380.1 |
| AtMYB123/TT2 | Chr05:13726743..13727961 | *Arabidopsis* | AT5G35550.1 |
| AtMYB5 | Chr03:4419960..4421751 | *Arabidopsis* | [AT3G13540.1](https://www.arabidopsis.org/servlets/TairObject?type=gene&id=40211) |
| AtMYB11 | Chr03:23154630..23156585 | *Arabidopsis* | [AT3G62610.1](https://www.arabidopsis.org/servlets/TairObject?type=gene&id=38235) |
| AtMYB12 | Chr02:19476326..19479597 | *Arabidopsis* | [AT2G47460.1](https://www.arabidopsis.org/servlets/TairObject?id=34905&type=locus) |
| AtMYB111 | Chr05:19998823..20001572 | *Arabidopsis* | [AT5G49330.1](https://www.arabidopsis.org/servlets/TairObject?id=132181&type=locus) |

**Supporting Information Table S4** Constructs used in this study.

| **Number** | **Gene** | **Vector** | **Origin** | **Remark** |
| --- | --- | --- | --- | --- |
| 18 | *ASR2*^inf^ (cDNA) | pDONOR | *P. inflata* | No coding |
| 68 | *ASR1*^inf^ (cDNA) | pDONOR | *P. inflata* | No coding |
| 70-1 | *ASR1*^M1^(cDNA) | pDONOR | M1 | Coding |
| 70-2 | *ASR1*^V30^(cDNA) | pDONOR | V30 | Coding |
| 72 | *ASR1*^inf^(DNA) | pDONOR | *P. inflata* | No coding |
| 73 | *ASR1*^inf^ (DNA) | OE | *P. inflata* | made from 72 |
| 75 | *ASR2*^M1^(cDNA) | pDONOR | M1 | Coding |
| 76 | *ASR*2^M1^(cDNA) | OE | M1 | made from 75 |
| 78 | *ASR*2^M1^(DNA) | pDONOR | M1 | Coding |
| 79 | *ASR2*^M1^(DNA) | OE | M1 | made from 78 |
| 81 | *ASR2*^inf^(DNA) | pDONOR | *P. inflata* | No coding |
| 82 | *ASR2*^inf^(DNA) | OE | *P. inflata* | made from 81 |
| 84 | *ASR3*^V30^(DNA) | pDONOR | V30 | Coding |
| 85 | *ASR3*^V30^(DNA) | OE | V30 | made from 84 |
| 91 | *AN1* (TC) | pDONOR | M1×R27 | Coding |
| 93 | *AN11* (TC) | pDONOR | M1×R27 | Coding |
| 95 | *ASR* ^M1^(TC) | pDONOR | M1 | Coding |
| 97 | *AN1* | pGADT7/GW | M1×R27 | made from 91 |
| 100 | *AN11* | pGBKT7/GW | M1×R27 | made from 93 |
| 101 | *AN11* | pGADT7/GW | M1×R27 | made from 93 |
| 104 | *ASR2*^M1^ | pGBKT7/GW | M1 | made from 95 |
| 105 | *ASR2*^M1^ | pGADT7/GW | M1 | made from 95 |
| 106 | *ASR3*^inf^(DNA) | pDONOR | *P. inflata* | Coding |
| 108 | *ASR2*^inf^ | OE | *P. inflata* | made from 18 |
| 109 | *ASR3*^M1^(cDNA) | pDONOR | M1 | Coding |
| 110 | *ASR3*^M1^(cDNA) | OE | M1 | made from 109 |
| 112 | *ASR1*^V30^(cDNA) | OE | V30 | made from 70-2 |
| 113 | *ASR1*^inf^(cDNA) | OE | *P. inflata* | No coding |
| 118 | *ASR3*^M1^(TC) | pDONOR | M1 | Coding (cDNA) |
| 119 | *ASR3*^M1^ | pGBKT7/GW | M1 | made from 118 |
| 120 | *ASR3*^M1^ | pGADT7/GW | M1 | made from 118 |
| 121 | *ASR1*^M1^(TC) | pDONOR | M1 | Coding |
| 122 | *ASR1*^M1^ | pGBKT7/GW | M1 | made from 121 |
| 123 | *ASR1*^M1^ | pGADT7/GW | M1 | made from 121 |
| 135 | pASR1^inf^ | pDONOR | *P. inflata* |  |
| 136 | pASR2^inf^ | pDONOR | *P. inflata* |  |
| 137 | pASR3^inf^ | pDONOR | *P. inflata* |  |
| 138 | pASR1^inf^ | Promoter | *P. inflata* | made from 135 |
| 139 | pASR2^inf^ | Promoter | *P. inflata* | made from 136 |
| 140 | pASR3^inf^ | Promoter | *P. inflata* | made from 137 |
| 151 | pASR1^M1^ | pDONOR | M1 |  |
| 152 | pASR1^M1^ | Promoter | M1 | made from 151 |

**Supporting Information Table S5** Primer sequences.

| **qRT-PCR primers** | **Sequence(5'-3')** |
| --- | --- |
| 523-*actin*-QF | AGCCAACAGAGAGAAGATGACCCA |
| 524-*actin*-QR | ACACCATCACCAGAGTCCAACACA |
| 525-*DFR*-QF | TGGAACTGCCAAAGGCTGATA |
| 526-*DFR*-QR | CTCCTTGACAGCCTTGAATGG |
| 527-*CHSaI*-QF | CAACTAGTGGTGTGGACATGCC |
| 528-*CHSaI*-QR | CACCAGCAAAGCAACCTTGTT |
| 533-*F3'H*-QF | AGCTGGACGTAGGATTTGTG |
| 534-*F3'H*-QR | ATGGATCAGCCCGTTGTAAG |
| 535-*AN2*-QF | GATGGACTTCAATGGTGGGCCAAT |
| 536-*AN2*-QR | CGATGGTGCTGTTTCCTCATGCAA |
| 537-*DPL*-QF | GAACGGCAAACGATGTGAAAAACTACTG |
| 538-*DPL*-QR | GCATTCTTGGCCATGGTCCTAATTTC |
| 539-*AN4*-QF | GGTGGTCACTTATTGCTGGGAG |
| 540-*AN4*-QR | TTGAGAGGTTCCGAGGTTGAGG |
| 541-*actin*-QF | CCTGATGAAGATCCTCACCGA |
| 542-*actin*-QR | CAAGAGCCACATAGGCAAGCT |
| 543-*ANS*-QF | TCTTCCATTGTGCTTTCCCTG |
| 544-*ANS*-QR | GTTGCTGGAGTGTAGTCAGTAG |
| 545-*PHZ*-QF | TAGCTAACTGCAACGAAAATGATGAAACA |
| 546-*PHZ*-QR | TCCTCGTGCCAACAAACTCGTAACTT |
| 547- *F3'5'H-1*-QF | ATAGGCGTTTACTCGAATCCG |
| 548- *F3'5'H-1*-QR | TGGTGTAGAAGGGTGTTTTCG |
| 549-*AT*-QF | AACCAAGGCTCGTAACACTC |
| 550-*AT*-QR | GGAGAGTGATTGGAATGTGGAG |
| 551-*DFR-*QF | CCCCTAGTTTAATCACTGCCC |
| 552-*DFR*-QR | GACCATCTTAGCCACATCGTAG |
| 563-*MT2*-QF | GCAGCGAGCTTTATGGGTCTT |
| 564-*MT2*-QR | TGAGCTTTAGGAACATCGATAGAAAC |
| 565-*MF1*+*MF2*-QF | CTTTTGGCTACTGCTCTTGCATT |
| 566-*MF1*+*MF2*-QR | CTCTGTCCGGATCAATCGCTAT |
| 581-*GST*-QF | GGTGACCAAGAGAAAGTGTTTGC |
| 582-*GST*-QR | GCTGGGATAGACACTGCTTCA |
| 583-*RT*-QF | CAGGGCTTCCTTTCTTTCTTGTCT |
| 584-*RT*-QR | TCCCCTTGTCTTTCACTCTTTCC |
| 590-*FLS*-QF | AAGGAAGTGGAAGGCAAGAAAGG |
| 591-*FLS*-QR | TCAAGCCCAAGCCCAAGAGA |
| 592-*LAR*-QF | AGCCTACTTTGTGTCTGGTCCT |
| 593-*LAR*-QR | GTGTTCTGCCAATCTTAGTTTCCC |
| 594-*ANR*-QF | TCTGGACGATACATTTGCTCTGC |
| 595-*ANR*-QR | AAACCCGTAGGAACATCTGAAGC |
| 596-*MYBFL*-QF | CGGACGAACTGACAACGAGATAA |
| 597-*MYBFL*-QR | CTGTGGTAGTGGTGGTGGTATTG |
| 645-*ANS*-QF | CAAGAGTTGAAGAAAGCAGCC |
| 646-*ANS*-QR | ACACACTTAGCAGTTACCCAC |
| 651-*3GT*-QF | CTACAATGTTTGGGATGGTGTC |
| 652-*3GT*-QR | TCTAATGCTTCTGCCACTGC |
| 653-*5GT*-QF | CCGTTGATTCCTTCTGCTTTC |
| 654-*5GT*-QR | GATCCACCTTCCTTCACAGC |
| 659-*ASRs*-QF | TGGTGAAGGAAAGTGGCATCTTG |
| 660-*ASRs*-QR | GGAAGTCTACCCGCAATAAGTGAC |
| 676-*C4Ha*-QF | GCAAATCACTGAGCCAGACACA |
| 677-*C4Ha*-QR | TAGCCAGCCACCAAGCGTTT |
| 678-*4CLa*-QF | GCAAGCACAATAGACAAAGATGGG |
| 679-*4CLa*-QR | ACTGGAACTTCTCCTGCTTGTTC |
| 680-*CHSj*-QF | GAGTCCTTGTTGTTTGTTCGGAG |
| 681-*CHSj*-QR | GAGAAGAGTTTGGCTTGCGGA |
| 682-*CoA*-QF | TGATTTGGCTGATGTATTTCCACC |
| 683-*CoA*-QF | TGATTTGGCTGATGTATTTCCACC |
| 694-*PALa*-QF | GCTAGGCGGTGAGACGCTAA |
| 695-*PALa*-QR | CTCGGACAGCTGCACTGTCA |
| 698-*F3H*-QF | CTGGTGGCAAGAAAGGTGGATT |
| 699-*F3H*-QR | GAGTAGTCTCTTGCCCTTGTTGG |
| 700-*CHIa*-QF | TCTCCTCCAGTGTCCGTTACTAAA |
| 701-*CHIa*-QR | ACTTCCCTTCTATCTCCAGACCTC |
| 704-*PH1*-QF | TCTTCATAAGTGTCTCTCTCCGCT |
| 705-*PH1*-QR | TAGTAGCCTCACATCACCTGGG |
| 706-*PH5*-QF | TGACAAGACAGGGACGCTAACC |
| 707-*PH5*-QR | TGGCAGCAAGTAACACAACCAT |
| 743- *F3'5'H-2*-QF | TGGAAACAATCGGCGTCTGC |
| 744- *F3'5'H-2*-QF | TGGTGTAGAAGGGTGCTTTCG |
| ***ASR* gene cloning primers** | |
| 82-*ASR1*-Fw | GGGGACAAGTTTGTACAAAAAAGCAGGCTATGAATACTATTGTTATCTGTAC |
| 83-*ASR1*-Rv (with stop codon) | GGGGACCACTTTGTACAAGAAAGCTGGGTCTAATTAAGAAGACTCCAGAGGTCA |
| 84-*ASR2*-Fw | GGGGACAAGTTTGTACAAAAAAGCAGGCTATGAATACTATTCCTAT |
|  | CTGTAC |
| 85-*ASR2*-Rv (with stop codon) | GGGGACCACTTTGTACAAGAAAGCTGGGTCTAATTAAGTAGACTCCAGAGG |
| 86-*ASR3*-Fw | GGGGACAAGTTTGTACAAAAAAGCAGGCTATGAATACAATTCCTATCTGTAC |
| 87-*ASR3*-Rv (with stop codon) | GGGGACCACTTTGTACAAGAAAGCTGGGTATTAAGTAGACTCCAGAGG |
| 83/2-*ASR1*-Rv (without stop codon) | GGGGACCACTTTGTACAAGAAAGCTGGGTATTAAGAAGACTCCAGAGGTCA |
| 85/2-*ASR2*-Rv (without stop codon) | GGGGACCACTTTGTACAAGAAAGCTGGGTATTAAGTAGACTCCAGAGG |
| 87/2-*ASR3*-Rv (without stop codon) | GGGGACCACTTTGTACAAGAAAGCTGGGTATTAAGTAGACTCCAGAGG |
| 82/2-*ASR1*-Fw | GGGGACAAGTTTGTACAAAAAAGCAGGCTTCATGAATACTATTGTTATCTGTAC |
| 84/2-*ASR2*-Fw | GGGGACAAGTTTGTACAAAAAAGCAGGCTTCATGAATACTATTCCTATCTGTAC |
| 86/2-*ASR3*-Fw | GGGGACAAGTTTGTACAAAAAAGCAGGCTTCATGAATACAATTCCTATCTGTAC |
| **ASR promoter primers** | |
| 325-pASR1^inf^-Fw | GGGGACAAGTTTGTACAAAAAAGCAGGCTACCATCTTCTCATTATTCCGAG |
| 323-pASR1^inf^-Rv | GGGGACCACTTTGTACAAGAAAGCTGGGTGCCAAAACCTAGCAATGATTG |
| 404-pASR2^inf^-Fw | GGGGACAAGTTTGTACAAAAAAGCAGGCTATCGCTCTGATTGCATTTCTG |
| 321-pASR2^inf^-Rv | GGGGACCACTTTGTACAAGAAAGCTGGGTGCTGCAAAAAATACCAGCTTAG |
| 416-pASR3^inf^-Fw | GGGGACAAGTTTGTACAAAAAAGCAGGCTCAGCGAAGGGAAATTAGGTTG |
| 317-pASR3^inf^-Rv | GGGGACCACTTTGTACAAGAAAGCTGGGTCCTCGGAAGCTGCAAATAGTAC |
| **Genome Walking primers** | |
| 586-pASR1^M1^-SP1 | GCCTCCCACAGTCGCAACTACTTACA |
| 587-pASR1^M1^-SP2 | ACTCCGCCAATAGTGGTCACTTGCAT |
| 589-pASR1^M1^-SP3 | GCGTCTGAAGCTGCCAAAACCTAGC |
| **PCR and RT-PCR primers** | |
| *ASR1/2/3*-F | CAGACGCCCATATCATGAATAC |
| 342-*ASR3*-R | GTTGATCTTACAGAAAGCTGTGC |
| 343-*ASR1*-R | ATTACATGAATTCGAAGGTATGC |
| 345-*ASR2*-R | CACTGGTCTCCATTATCTGTTG |
| 346-*ASR1*-R | CATTGAACTCCATTTTCTGTCG |
| 465-*ASR1*-F | TCCTTCTCCATCGCATCCTC |
| 219- *actin-F* | CTACGAGGGTTATGCTTTGCC |
| 220- *actin-R* | GCTGGAATGTGCTAAGGGATG |
| 284-*AN2*-Fw | GCTTCATAAGCTTCTAGGCAAC |
| 285-*AN2*-Rv | TAAGGCTGCTTTCAGCATTTAC |
| 750-*F3'5'H-1*-F | ATGATGCTACTTACTGAGCTTGC |
| 746- *F3'5'H-1*-R | GGCCCTCTCGACTCATATCGG |
| 3810- *F3’5’H-1*-F | CCAAATCTCCCTTACCTCC |
| 726-*F3’5’H-1*-R | CATCTATAGCTATGGTACATAAAC |
