# Supplementary material for: Identification and functional analysis of three new anthocyanin R2R3‐MYB genes in Petunia
Source: Plant Direct. 2019 Jan 21;3(1):e00114. doi: 10.1002/pld3.114 (PMC6508765; doi:10.1002/pld3.114)
Supplement: Supplementary file 3 [file PLD3-3-e00114-s003.pdf]

Dear editor,

Thanks for giving us the second time to improve our manuscript. Based on your suggestions, we have asked one English language editing service providers to edit it.

We summarize here below the changes and we uploaded a file of the manuscript containing the track changes marking:

Comments of reviewers

1. I have requested in my previous decision letter that this manuscript goes through English language editing. Although the writing has been improved, many grammar mistakes are still present through out the manuscript. Please seek professional English language editing assistance like the one offered by Wiley (https://wileyeditingservices.com/en/english-language-editing/). I list a few mistakes I found below:

Line 50: inactivated by two different single base insertions in their coding sequence. All three these genes

All three "of" these genes

Line 66: ASR3 is only weak ("weakly") expressed in stigma

Line 206: This brings up the previously proposed possibility that other wild petunia species, next to P. inflata and P. axillaris, contributed......

When you say "next to", do you mean "in addition to"?

Line 342: how they diverged during evolution is an important question in both Developmental and Evolution Biology (developmental and evolution biology).

【Answer】Thanks for pointing out these detailed questions for us. We have corrected all these points.

2. Plant Direct do not allow statements supported by "data now shown". Please remove all following sentences completely.

Line 235: (ASR2M1-OE, ASR3M1-OE and ASR3inf-OE, similar phenotype with ASR1M1-OE; data not shown) were pigmented, while this was not the case for ASR1inf-OE, (ASR2inf-OE, similar phenotype, data now shown)

Line 248: We could not measure obvious differences in the pH of the crude petal extract for flowers of any of the ASRM1-OE lines when compared to untransformed controls, suggesting that ASRs do not regulate the structural genes involved in vacuolar hyper-acidification (Faraco et al., 2014).

【Answer】We have deleted the sentences or added some more figures to support these data (Figure3). For line 248, the supporting data have been shown in figure 3. Please check the petal picture on the right of each panel (after bud opening).

3. I have asked in my previous decision letter for "Supplemental Figure 4B, please label

clearly which ASR gene was analyzed" and this is still not corrected. There is only one bar for ASR so it cannot be "ASR1 and ASR2" or "ASRs". It can only be one gene.

【Answer】The sequences of ASRs were too similar. No suitable specific primers can be used for qRT-PCR. The ASR MYBs primers (559 and 560) we used can amplify transcripts from both of *ASR1* and *ASR2*, therefore the graph shows the total expression of them.
Sorry to confuse you here.
We have made this clear now in the text and in the legends of Supplemental Figure 4. Many thanks.

We hope this manuscript has definitely improved after this revision and we are grateful to the reviewers for your suggestions. We hope that you will now consider the manuscript suitable for publication in Plant Direct.

We are eager to hear from you
Best regards
Hechen Zhang and Francesca M. Quattrocchio
